# Environmentally Relevant Iron Oxide Nanoparticles Produce Limited Acute Pulmonary Effects in Rats at Realistic Exposure Levels

**DOI:** 10.3390/ijms22020556

**Published:** 2021-01-08

**Authors:** Chang Guo, Ralf J. M. Weber, Alison Buckley, Julie Mazzolini, Sarah Robertson, Juana Maria Delgado-Saborit, Joshua Z. Rappoport, James Warren, Alan Hodgson, Paul Sanderson, James Kevin Chipman, Mark R. Viant, Rachel Smith

**Affiliations:** 1 Centre for Radiation, Chemical and Environmental Hazards, Public Health England, Harwell Campus, Didcot, Oxfordshire OX11 0RQ, UK; alison.buckley@phe.gov.uk (A.B.); sarahb.robertson@phe.gov.uk (S.R.); james.warren@phe.gov.uk (J.W.); alan.hodgson@phe.gov.uk (A.H.); 2 School of Biosciences, University of Birmingham, Birmingham B15 2TT, UK; r.j.weber@bham.ac.uk (R.J.M.W.); j.k.chipman@bham.ac.uk (J.K.C.); m.viant@bham.ac.uk (M.R.V.); 3 Centre for Discovery Brain Sciences, University of Edinburgh, Edinburgh EH16 4SB, UK; julie.mazzolini@ed.ac.uk; 4 School of Geography, Earth and Environmental Sciences, University of Birmingham, Birmingham B15 2TT, UK; delgado@uji.es (J.M.D.-S.); psanderson@wardell-armstrong.com (P.S.); 5 School of Medicine, Environmental Health and Clinical Research, Universitat Jaume I, Perinatal Epidemiology, 12071 Castellon, Spain; 6 ISGlobal Barcelona Institute for Global Health, Barcelona Biomedical Research Park, 08003 Barcelona, Spain; 7 Environmental Research Group, MRC Centre for Environment and Health, Imperial College London, London W12 7TA, UK; 8 Department of Biology, Boston College, 140 Commonwealth Avenue, Chestnut Hill, MA 02467, USA; rappopoj@bc.edu; 9 Wardell Armstrong, Sir Henry Doulton House, Forge Lane, Stoke-on-Trent ST1 5BD, UK

**Keywords:** iron oxide, nanoparticle, ultrafine, inhalation, lung, rat, omics

## Abstract

Iron is typically the dominant metal in the ultrafine fraction of airborne particulate matter. Various studies have investigated the toxicity of inhaled nano-sized iron oxide particles (FeO_x_NPs) but their results have been contradictory, with some indicating no or minor effects and others finding effects including oxidative stress and inflammation. Most studies, however, did not use materials reflecting the characteristics of FeO_x_NPs present in the environment. We, therefore, analysed the potential toxicity of FeO_x_NPs of different forms (Fe_3_O_4_, α-Fe_2_O_3_ and γ-Fe_2_O_3_) reflecting the characteristics of high iron content nano-sized particles sampled from the environment, both individually and in a mixture (FeO_x_-mix). A preliminary in vitro study indicated Fe_3_O_4_ and FeO_x_-mix were more cytotoxic than either form of Fe_2_O_3_ in human bronchial epithelial cells (BEAS-2B). Follow-up in vitro (0.003, 0.03, 0.3 µg/mL, 24 h) and in vivo (Sprague–Dawley rats, nose-only exposure, 50 µg/m^3^ and 500 µg/m^3^, 3 h/d × 3 d) studies therefore focused on these materials. Experiments in vitro explored responses at the molecular level via multi-omics analyses at concentrations below those at which significant cytotoxicity was evident to avoid detection of responses secondary to toxicity. Inhalation experiments used aerosol concentrations chosen to produce similar levels of particle deposition on the airway surface as were delivered in vitro. These were markedly higher than environmental concentrations. No clinical signs of toxicity were seen nor effects on BALF cell counts or LDH levels. There were also no significant changes in transcriptomic or metabolomic responses in lung or BEAS-2B cells to suggest adverse effects.

## 1. Introduction

Air pollution is a complex, multi-source mixture of gases and particulate matter, containing organic and inorganic compounds, and is a significant cause of ill health worldwide [1]. Despite the wealth of epidemiological evidence for the overall effects of air pollution, it remains unclear which components contribute to the various effects on health [2,3] or indeed whether these are driven by synergistic effects between the various pollutants. One focus of such studies has been on the ultrafine (nano-sized, i.e., <100 nm) particulate fraction [4], as it has been hypothesised that the greater number of particles and larger surface area per unit mass of this fraction, in comparison to larger particles, might result in increased biological activity through, for example, enhanced generation of reactive oxygen species [5] or greater potential for cellular uptake [6]. The main source of ultrafine particles is road traffic, although other forms of transport (e.g., ships), industrial activities and natural sources (e.g., in marine environments) also contribute [7]. The ultrafine fraction includes a range of particle types including some that are predominantly metallic. The main sources of metal emissions to air in the UK include metal smelting and refining processes, as well as transport and domestic biomass burning [8], and the most abundant metal is iron, with concentrations typically representing up to 1% of the aerosol mass and fluctuating between 400 and 600 ng/m^3^ in the last two decades [8]. Higher concentrations have been reported in some locations, including the London Underground where iron has been found to comprise greater than 40% by mass of PM_2.5_ [9,10,11,12]. Iron is also the dominant metal in the ultrafine fraction in the UK [13] and other countries, including urban settings in China [14] and Mexico [15]. The main sources of this iron are road vehicles and originate from brake wear and other vehicle erosion particles and from trace quantities in fuels and lubricating oils [13]. In a companion study to that reported here, submicron atmospheric particles (<180 nm) at two UK urban sites (roadsides in Birmingham and Newcastle) were collected and characterised. Iron oxide particles with different physical and chemical properties were found to be the most abundant metallic particles collected, including particles of FeO, Fe_3_O_4_, α-Fe_2_O_3_ and γ-Fe_2_O_3_, of which γ-Fe_2_O_3_ was the most numerous [16]. Overall, the iron content of the ultrafine fraction (particles < 100 nm) was in the range 10–100 ng/m^3^ [16]. Detailed analysis of particles sampled from these locations found atmospheric submicrometer particles (<180 nm) included two types of iron containing particles, defined as high iron content (ca 90%, other elements <1%) and moderate iron content (ca 75%, with typically high manganese and silicon content). Primary particles of both were of spherical or near spherical morphology with sizes in the range 15–80 nm (median 30–40 nm). These findings were consistent with the size of spherical iron oxide nanoparticles (FeO_x_NPs) in the range 10 to 100 nm measured from samples collected in Tokyo [17]. The primary particles existed in agglomerates of typically a few hundred nm (range 200 nm to 1 µm) [16]. The presence of iron-based agglomerates of this type has been identified previously [18]. The oxidation state of the iron was investigated, and primary particle size was found to vary with chemical form with median diameters of 27 ± 21, 13 ± 12 and 32 ± 22 nm, respectively, for Fe_3_O_4_, α-Fe_2_O_3_ and γ-Fe_2_O_3_. Furthermore, Sanderson et al. [16] estimated that the % distribution (by number) of nano-sized primary particles of the three main iron oxides observed in the ultrafine fraction of the roadside aerosol were as follows: 32% Fe_3_O_4_, 44% γ-Fe_2_O_3_ and 20% α-Fe_2_O_3_.

A number of studies (both in vitro and in vivo (Table S1)) have been undertaken to investigate the toxicity of inhaled nano-sized iron particles or iron-containing ambient particulates (e.g., iron-soot). Exposure to iron nanoparticles at the air-liquid interface has shown cellular toxic effects including oxidative stress [19,20] and DNA damage [21]. Deposition of inhaled iron particles has mainly been found in the alveolar region [22,23,24], with some distribution to various organs including the liver, testis, spleen and brain [23,25]. A recent whole-body inhalation exposure study using iron-soot combustion particles (Count median diameter (CMD) 50.4 ± 4 nm) found that iron NPs were transported to the brain via the olfactory nerves and were associated with indicators of neural inflammation [26]. A few inhalation studies assessing pulmonary toxicological effects have reported oxidative stress, macrophage infiltration and inflammation [27,28,29,30,31], although none, even those using repeated high exposure doses, reported effects on breathing rate or mortality. In contrast, some other studies reported little if any pulmonary effects [25,32,33,34]. The majority of these studies, many of which were focussed on the safety of manufactured nanomaterials, also did not use materials reflecting the characteristics of FeO_x_NPs particles present in the environment.

The presence of FeO_x_NPs in ambient air, the existence of contradictory results from previous inhalation studies of FeO_x_NPs in the literature and the lack of studies using environmentally relevant particles prompted this study, the purpose of which was to analyse the potential toxicity of FeO_x_NPs with environmentally relevant characteristics, applying both in vitro and in vivo experimental models. The study used FeO_x_NPs of different forms manufactured to match the characteristics of ‘High Fe content’ nano-sized particles sampled from the environment, both individually and in an environmentally representative mixture (FeO_x_-mix). A study addressing this area is considered particularly pertinent given the importance of metals, including iron, within non-exhaust sources of pollutants such as brake and engine wear, which are of growing importance given the increasing focus on electric vehicles [35,36].

**Figure 1:**
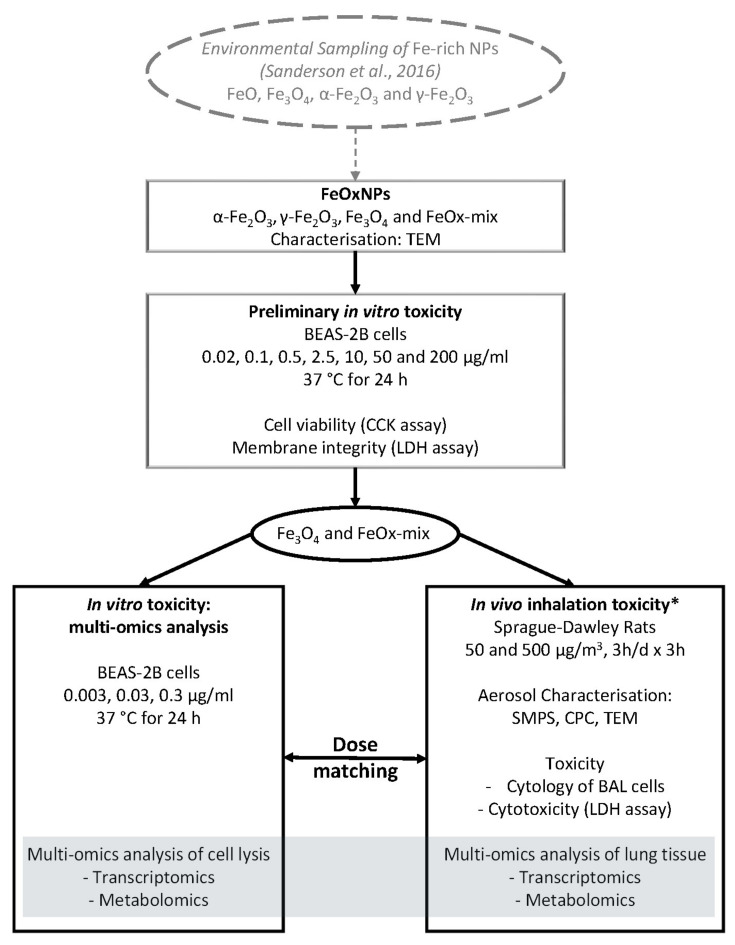
Pulmonary toxicity of environmentally relevant FeO_x_NPs: Study design. (* additional in vivo inhalation study was undertaken to investigate effects on Heart Rate Variability (HRV), see text for details).

Both human pulmonary epithelial cells (BEAS-2B) and animals (Sprague–Dawley rats) were exposed to FeO_x_NPs relevant to those found environmentally, and assessed by parallel approaches including detailed particle characterisation, toxicity assessment and multi-omics analyses (Figure 1). Preliminary in vitro experiments characterised the cytotoxic potential over a wide dose range of both the environmentally relevant mix (FeO_x_-mix) and the different component particles to prioritise materials for further study. Further in vitro experiments were then undertaken using these materials to explore responses at the molecular level via multi-omics analyses at dose levels below those at which significant cytotoxicity was evident. In parallel, to explore effects on the whole lung, in vivo inhalation experiments were undertaken using aerosol concentrations chosen on the basis of modelling results to produce similar levels of particle deposition on the airway surface as were delivered to the in vitro cell cultures (dose matching). As part of this study, preliminary experiments were also undertaken to investigate the potential effects of exposure to a FeO_x_NP aerosol on Heart Rate Variability (HRV) using the same exposure system with aged animals and a novel non-implant telemetry system (Supplementary Information 1).

## 2. Results

### 2.1. Toxicity Assessment of FeO_x_NPs in BEAS-2B Cells

#### 2.1.1. Characterisation of FeO_x_NPs

Analysis of TEM images of the α-Fe_2_O_3_, γ-Fe_2_O_3_ and Fe_3_O_4_ NPs indicated mean primary particle sizes of 11 ± 9 nm, 5 ± 2 nm and 19 ± 16 nm, respectively (Table 1). These are smaller than those identified in the environment [16], but the difference is only statistically significant for γ-Fe_2_O_3_ (independent sample *t*-test, *p* < 0.001). The shape of the FeO_x_NPs were variable. Individual α-Fe_2_O_3_ and Fe_3_O_4_ NPs were typically spherical, whereas γ-Fe_2_O_3_ NPs were more elongated (Figure S2).

#### 2.1.2. Cytotoxic Effects of FeO_x_NPs on Human Bronchial Epithelial Cells (BEAS-2B)

**Figure 2:**
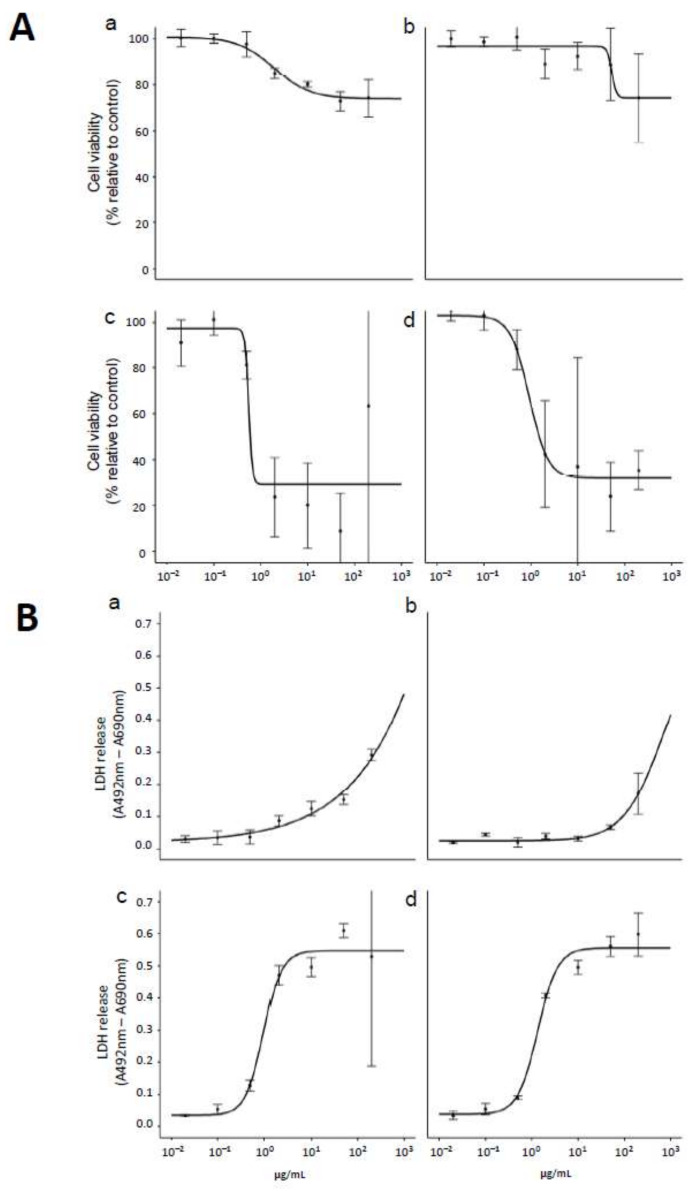
Effects of FeO_x_NPs on BEAS-2B cell viability and membrane integrity. Cells were treated with γ-Fe_2_O_3_ (a), α-Fe_2_O_3_ (b), Fe_3_O_4_ (c) and FeO_x_-mix (d) in a range of concentrations from 0.02 to 200 g/mL in SCM for 24 h at 37 °C then (**A**) cell viability was measured using CCK-8 assay and (**B**) membrane integrity was measured by lactate dehydrogenase (LDH) release assay. Data represent means and SD of three technical replicates.

The effects of FeO_x_NPs on the viability and membrane integrity of BEAS-2B cells were tested using CCK-8 and Lactate Dehydrogenase (LDH) release assays, respectively. As observed in Figure 2, α-Fe_2_O_3_ and γ-Fe_2_O_3_ treatments had only a minor effect on cell viability and LDH release (Figure 2B) at relatively low concentrations but a trend of increasing LDH release started around 1–5 μg/mL. Interestingly, cell treatment with Fe_3_O_4_ and FeO_x_-mix produced more marked effects with strong decreases in cell viability, reaching a plateau around 25% viability and with ED_50_ values of 0.59 ± 0.90 μg/mL and 0.91 ± 0.17 μg/mL, respectively (Figure 2A). These observations were supported by LDH release measurements (Figure 2B), which showed large increases, reaching a plateau around 0.5–0.6 (a.u.), with ED_50_ = 0.96 ± 0.20 μg/mL for Fe_3_O_4_ and ED_50_ = 1.34 ± 0.21 μg/mL for the FeO_x_-mix. Thus, these results indicate that oxide form influenced the cytotoxicity of FeO_x_NPs in serum-containing media on BEAS-2B cells, with Fe_3_O_4_ having a greater effect than either form of Fe_2_O_3_, and the effect of the mix was dominated by Fe_3_O_4_.

#### 2.1.3. Multi-Omics Analysis of BEAS-2B Cells

**Figure 3:**
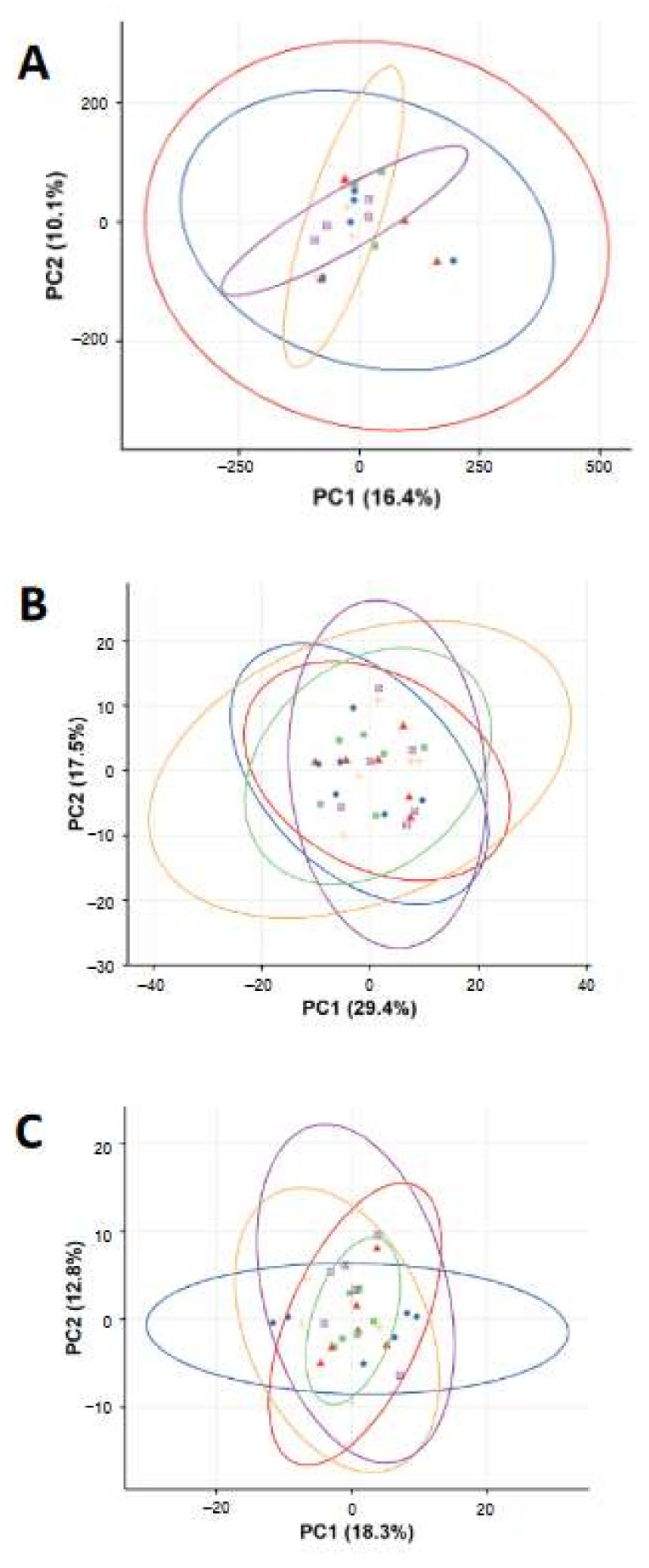
Principal component analysis (PCA) score plots of the gene expression (**A**) and metabolomics profiles ((**B**) in positive ion mode and (**C**) in negative ion mode) of BEAS-2B cells treated with H_2_O and FeO_x_NPs. Symbols in the PCA plots indicate individual repeats of BEA-2B cells of control group (circle 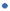) or exposed to Fe_3_O_4_ B (triangle 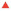), FeO_x_-mix A (square 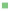), FeO_x_-mix B (plus 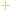), and FeO_x_-mix C (plus 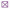).

The biological effects of FeO_x_NPs (Fe_3_O_4_ and FeO_x_-mix) on BEAS-2B cells were further analysed using higher information content techniques, including global gene expression by microarray analysis and identification of changes in small molecule substrates by metabolomics analysis, at exposure levels that the preliminary study had shown to be sub- or low- cytotoxic. This was to ensure that responses observed would reflect exposures rather than being dominated by secondary effects related to cell death. Principal component analysis (PCA) score plots from the analysis of the gene expression and metabolomics measurements are shown in Figure 3. For the gene expression analysis, there was no clear visual separation between any groups, either from comparison at the same concentration of FeO_x_-mix NPs vs. Fe_3_O_4_ NPs (Figure S3A) or comparison of different concentrations of FeO_x_-mix NPs (Figure S3B). For the metabolomics data, there was no clear visual separation between FeO_x_NPs exposed groups and control groups (Figure 3B,C) for both polar (positive ion mode) and non-polar (negative ion mode) assays, neither for the comparison at the same concentration of FeO_x_-mix NPs and Fe_3_O_4_ NPs (Figure S3C and Figure 3E) nor for the comparison at different concentration of FeO_x_-mix NPs (Figure S3D and Figure 3F). There were very few differentially (q-value < 0.05 and log2 fold change > 1.0) expressed genes (n < 5) and metabolite features (q value <0.05) that could be identified as significant from any multi-group comparisons. Taken together, multi-omics analysis of BEAS-2B cell lysates indicated no significant biological effects induced by FeO_x_NPs at the examined concentrations.

### 2.2. Nose-Only Inhalation Study of FeO_x_NPs in Sprague Dawley Rats

#### 2.2.1. Characterisation of FeO_x_NP Aerosols and Deposited Doses

**Figure 4:**
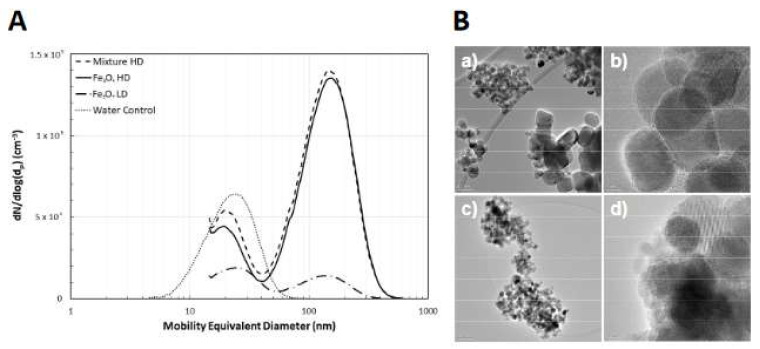
Aerosol characterisation. Number-weighted mobility equivalent particle size distributions averaged over the entire exposure duration for each aerosol exposure group (**A**). Error bars are not shown for clarity. Representative TEM images of the aerosol particles (**B**) delivered to the Fe_3_O_4_ high-dose (a,b) and FeO_x_-mix high-dose (c,d) exposure groups. The scale bars are as follows: (a) 50 nm; (c) 100 nm; (b) and (d) 5 nm.

Results of the aerosol characterisation are presented in Figure 4 and Table 2. Figure 4B shows representative TEM images of the iron oxide aerosol particles. For comparison, images of the iron-rich atmospheric aerosol particle sampled from a roadside location can be found in Sanderson et al. [16]. Average primary particle sizes, measured from the TEM images (n = 140), were 19 ± 13 nm and 18 ± 10 nm for the Fe_3_O_4_ and FeO_x_-mix aerosols respectively, consistent with the suspension particle sizes (Table 1). Aerosol size distributions averaged over each exposure group are given in Figure 4A. Two peaks can be observed in the size distribution, the larger (~150 nm) consisting of the FeO_x_NPs and the smaller (~20nm) a residue peak, formed from impurities in the water and leaching from the walls of the container and is an unavoidable consequence of the nebulization process [37,38]. Note that for the control exposures, only the residue peak is observed. Table 2 summarises the measured aerosol characteristics for the iron-oxide peaks only (additional aerosol metrics Table S3).

Deposited mass dose estimates for the in vitro and in vivo exposures are summarised in Table 3 (for other dose metrics see Table S4). The deposited doses per unit tracheobronchial surface area for the two aerosol concentrations, 11 and 110 ng/cm^2^, respectively, are broadly similar to the doses per unit cell culture surface area, 9.9 and 99 ng/cm^2^, respectively, for the two higher in vitro concentrations, 0.03 and 0.3 µg/mL (equivalent aerosol concentrations of 44 and 440 µg/m^3^).

#### 2.2.2. Assessment of Gross Toxicity

**Figure 5:**
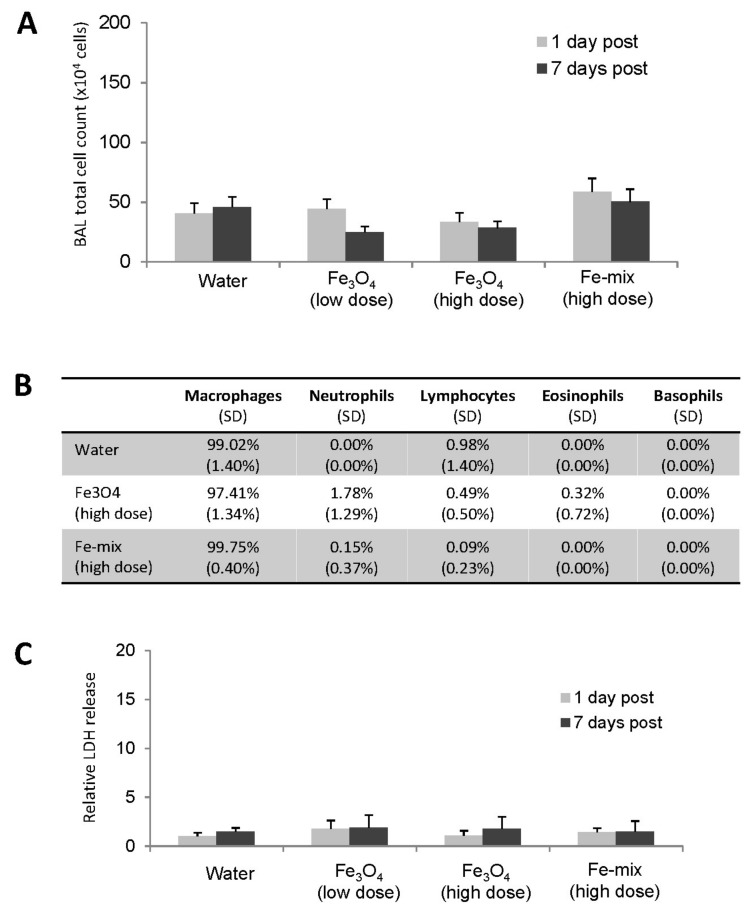
Cytological analysis of BALF. (**A**) Total cell counts of different exposure groups. (**B**) Cell population change at 1-day post exposure (SD values in brackets). (**C**) Gross toxicity analysis by LDH assay.

There were no adverse clinical signs observed in any of the experimental rats and no effects on their body weight development (data not shown). BALF analysis was undertaken at one day and seven days post exposure. There was no significant change to any exposure group in the total cell counts compared to the control group (Figure 5A). Cell population counting was also performed for the high concentration exposure groups at 1-day post exposure and the majority of the cells in the BALF from all experimental groups were macrophages (>97%) with no significant difference from controls. There were limited differences between control and exposed for other types of inflammatory cells including neutrophils, lymphocytes, eosinophils or basophils (Figure 5B). Further, lactate dehydrogenase (LDH) levels were measured but there was no significant change to any exposure group (Figure 5C). Consequently, there was no evidence of pulmonary toxicity from these measurements.

#### 2.2.3. Multi-Omics Analysis of Lung Tissues

**Figure 6:**
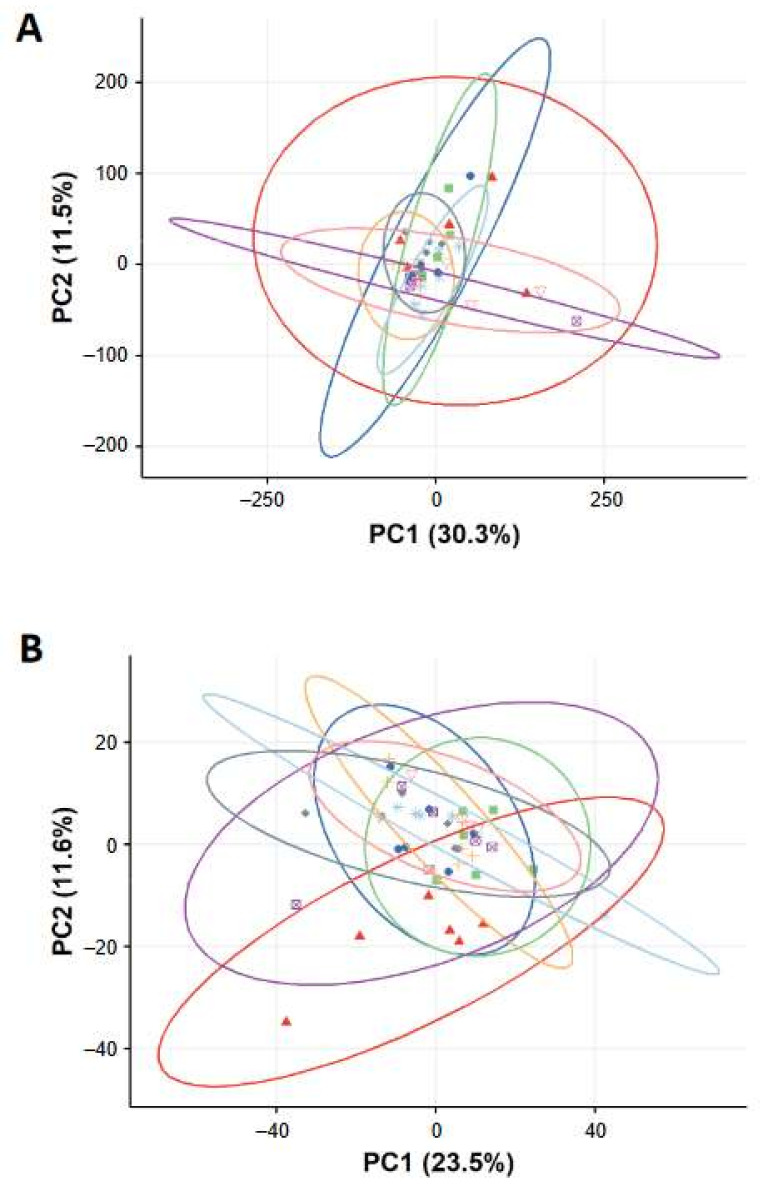
Principal component analysis (PCA) score plots of the gene expression (**A**) and metabolomics profiles (**B**) of lung tissue from rats exposed to control and FeO_x_NP aerosols. Symbols in the PCA plots indicate individual samples of rat lungs exposed to Water at one day (circle 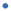) or 7 days (triangle 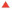) post-exposure, Fe_3_O_4_ (low dose) at one day (square 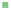) or seven days (plus 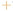) post-exposure, Fe_3_O_4_ (high dose) at one day (square with cross 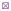) or seven days (astrix 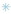) post-exposure, and FeO_x_-mix (high dose) at one day (diamond 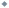) and seven days (inverted triangle 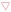) post-exposure.

Microarray analysis and metabolomics analysis were further performed to investigate any biological effects induced by the inhaled FeO_x_NPs in the lungs. Principal component analysis (PCA) score plots from both analyses are shown in Figure 6. For the gene expression analysis, there was no clear visual separation between any groups, for both one day and seven days post-exposure comparisons (Figure 6A, Figure S4A,B) and there were very few significantly differentially expressed genes (n < 5) for any of the multi-group comparisons. For the metabolomics data, when all groups were considered together, there was no clear visual separation between groups (Figure 6B). However, when considering seven-day post-exposure groups alone, the control group was separated from all other FeO_x_NP exposed groups (Figure S4D). This effect was not observed at one-day post-exposure (Figure S4C) and is complex to interpret (see Discussion). Overall, these results indicate limited adverse molecular responses and combined with the BALF results above support a conclusion of limited pulmonary toxicity of the FeO_x_NPs at the examined doses.

## 3. Discussion

The aim of this study was to explore the toxicity of inhaled FeO_x_NPs with environmentally relevant characteristics driven by the results of environmental sampling [16]. As a first step an in vitro study of the three principal iron oxide types and an environmentally relevant mix of them (FeO_x_-mix) was undertaken using a pulmonary epithelial model (BEAS-2B cells). The results indicated that both forms of Fe_2_O_3_ were less toxic than Fe_3_O_4_ and the FeO_x_-mix (the effect of which appeared to be dominated by the Fe_3_O_4_) (Figure 2). Few studies have explored the relative toxicity of different iron oxide forms but, consistent with our findings, Park et al. [39] demonstrated that Fe_3_O_4_NPs are more toxic than γ-Fe_2_O_3_NPs in murine alveolar macrophages and Pauluhn and Wiemann [33] reported a two-week inhalation study that found a consistent trend that rats exposed to Fe_3_O_4_ displayed more pronounced changes in BALF than those exposed to α-Fe_2_O_3_. It has been hypothesised that oxidative stress is the primary driver for the adverse effects of exposures to FeO_x_NPs and that this is heavily dependent on particle uptake, dissolution and release of free iron ions that can generate reactive oxygen species [40]. The importance of dissolution is supported by the results of Park et al., which demonstrate the greater toxicity of the more soluble Fe_3_O_4_. This is consistent with acellular studies that have demonstrated that Fe_3_O_4_NPs are more effective than γ-Fe_2_O_3_NPs in catalysing H_2_O_2_ production in acidic lysosomal conditions [41]. Other nanoparticle characteristics may also impact on their reactivity and thus toxicity. For example, studies have highlighted the potential importance of the particle size dependent Ce^3+^/Ce^4+^ surface speciation ratio of cerium dioxide nanoparticles on their reactivity and toxicity (e.g., Auffan et al. [42]). The effect of surface characteristics is yet to be directly studied for uncoated FeO_x_NPs of different isoforms, although studies have found that coatings can have a significant influence on chemical reactivity and ultimately toxicity [43,44]. In general, it is anticipated that the toxicity of FeO_x_NPs will depend upon a range of physico-chemical factors (e.g., size, shape, agglomeration status, solubility, dose etc.), which influence uptake of particles by cells and their chemical reactivity, including redox potential, and is worthy of further detailed study using well characterised materials, including a detailed assessment of potential contaminants.

Following this preliminary work, in vitro and in vivo studies were undertaken using the FeO_x_-mix and Fe_3_O_4_ alone, as this particular form appeared to play a key role in determining the in vitro cytotoxic effects of the mixture. For these two materials, significant changes in cell viability had been seen between 0.1 and 1.0 µg/mL (i.e., 10% EC_50_ ca 0.1 µg/mL and EC_10_ ca 0.3 µg/mL) and therefore concentrations for the further in vitro experiments were chosen to cover a range with low levels of cytotoxicity, i.e., 0.03 µg/mL (the maximum concentration before cytotoxicity was clearly evident) for both materials, with, in addition, a higher and lower concentration (0.3 and 0.003 µg/mL) for the FeO_x_-mix. These concentrations were chosen to ensure that the molecular responses observed would not simply be reflective of secondary changes resulting from cell death. An in vivo model was also used in parallel to investigate effects on the whole lung. Exposures for the in vivo study were ‘matched’ to those in vitro, i.e., chosen on the basis of modelling results to deliver mass doses per unit surface area in the tracheobronchial region broadly equivalent to the two higher in vitro doses (Table 3). The aerosol concentrations chosen on this basis significantly exceeded environmental levels (see later discussion). The results demonstrated low toxicity of the tested materials at the examined doses, as there was no evidence of significant molecular responses (gene or metabolite expression) in either the lung epithelial cell culture or rat lung model (Figure 3 and Figure 6) and no meaningful changes to inflammatory cell counts and LDH release in vivo (Figure 5).

The results from the current study are broadly consistent with some inhalation studies in the literature (Table S1). Following whole-body exposure to iron (γ-Fe_2_O_3_, 7.6 mg/m^3^) of similar size to the FeO_x_NPs in our study for 4 h (i.e., similar C X T ≈ 30 cf 5), C57Bl/6 mice showed no effects on BALF cells or LDH and following two weeks exposure (3.55 mg/m^3^ × 4 h/d × 5 d/w × 2 weeks) there was again no change in LDH levels although some inflammatory markers were significant immediately post-exposure but recovered after 3 weeks post exposure [32]. In one nose-only inhalation study using Wistar rats exposed to α-Fe_2_O_3_ (CMD 0.255 µm), no effects on BALF were reported even though the exposure dose was much higher (30 mg/m^3^ × 6 h/d × 5 d) [34] than that used here. In another short-term study (6 h/d × 3 d), Sprague–Dawley rats were exposed to γ-Fe_2_O_3_ NPs of larger primary particle size (72 nm) but similar aerosol particle size and concentrations (60 and 90 µm/m^3^) to our study, and no changes in LDH or BALF cell numbers were seen, although some measures of oxidative stress were significantly increased at the higher concentration, albeit at low levels [27]. However, other studies have found more significant effects, for example, in another nose-only inhalation study, Balb/c mice exposed to carboxylated Fe_3_O_4_NPs (CMD 68.6 nm, 19.9 mg/m^3^ × 4 h) showed significant inflammatory responses in the alveolar region immediately post-exposure and 815 genes significantly changed with the majority of up-regulated genes involved in immune response, chemotaxis and leukocyte activation, although gene expression changes in the primary chemokines were almost all resolved to control levels by 24 h post exposure [31]. Srinivas et al. [29] also found significant inflammatory effects following short term nose-only exposure to Fe_3_O_4_NPs (50 nm primary particle size, 640 mg/m^3^ × 4 h). Although it is difficult to directly compare the results of all these studies due to differences in material and exposure characteristics and overall study design, they suggest that the lack of significant effects on BALF parameters and gene expression observed in our study are possibly due to the lower levels of exposure (see also later discussion on dose metrics).

The authors are not aware of any earlier FeO_x_NP inhalation studies that have considered additional ‘omics’ analyses but Billing et al. [45] collected BALF at one and three-days post intratracheal instillation of Fe_3_O_4_NPs (<40 nm) in C57BL/6J mice and undertook proteome analysis by LC-MS/MS. Clustering of the differentially expressed proteins indicated an immune response, with the highest expression increase attributed to neutrophil extracellular trap formation. This contrasts with the limited metabolomic differences between exposed and control samples in the current study (Figure 6B). However, the doses were much higher than used here (i.e., 54 µg and 162 µg per mouse compared to 0.8 µg and 8 µg per rat), which resulted in significant neutrophil influx.

When all exposure groups are considered together the differences in gene expression and metabolites between exposed and control appear to show no evidence of responses that might indicate adverse effects (Figure 6), however, when the groups at one and seven days are considered separately, an unexpected pattern emerges. For gene expression there remains no clear visual separation between exposed and control groups at either time point (Figure S4A,B) with individual exposed vs. control group comparisons indicating few significantly differentially expressed genes (n < 5 in number of differentially expressed genes). However, for the metabolomics, although there is no clear separation at one-day post-exposure (Figure S4C), at seven days the control group demonstrates some separation from the exposed groups (Figure S4D). One interpretation is that this indicates an effect of exposure (possibly a delayed impact mediated by FeO_x_NP dissolution kinetics), which is relatively small and as such identified only by this relatively sensitive omics technique. However, the grouping of all exposed groups at one and seven days, including the lack of any apparent dose effect, complicates this initial interpretation. It seems unlikely that this effect arises from a design characteristic of the exposure system. One of our previous studies using the same exposure system with an almost identical protocol also found no separation between one- and seven- day post-exposure control groups on the basis of gene expression [46]. It is well known that restraint stress can affect animal heart rates, blood pressure and breathing rates, all of which tend to return to baseline within hours, which is one reason why acclimatisation activities are undertaken. In this context, the animals were observed throughout the exposures and showed no signs of distress. Although a number of in vivo studies have examined the effect of various stressors on the brain at a molecular level, effects on other organs including the lung have not been well studied, however Oishi and Machida [47] found changes in the mRNA expression of antioxidant enzymes in rat liver following 6h significant immobilisation stress, which decreased to baseline by 24 h, although no effects were seen in the heart, kidney or lung. It therefore seems unlikely that the effect seen relates to this factor. The addition of naive groups to the experimental design may have assisted in elucidating the origin of the effect and will be considered for future studies.

Toxic effects of inhaled FeO_x_NPs have been explored in other organs including the spleen, brain and heart. In one inhalation study, FeO_x_NPs were found to induce extramedullary hematopoiesis in the spleen although no effects on the pulmonary system or other organs [48]. The effects of adding iron to PM (in the form of iron-soot or soot) on heart rate variability (HRV) in mice were tested and it was found that inclusion of iron enhanced the PM_2.5_-exposure-reduced HRV [49]. Later, inhalation of Fe_2_O_3_ was shown to induce oxidative stress in the heart in compliance with increased ROS in the lung [30] and, more recently, iron-rich exogenous nanoparticles have been identified within human myocardial mitochondria and appear associated with mitochondrial dysfunction and oxidative stress [50]. As part of this study preliminary experiments were also undertaken to investigate the potential effects of exposure to an Fe_3_O_4_NP aerosol on HRV, as changes in this could be indicative of adverse cardiovascular health effects [51], using the same exposure system with older animals (18 months) and a novel non-implant telemetry system. This found no consistent effects of exposure on HRV, but due to the small sample size was of limited statistical power and thus it may be worth exploring further in the future. Details of these preliminary experiments can be found in Supplementary Information 1.

The development of appropriate in vitro models is a priority for toxicology. This process is aided by the demonstration of comparability between in vitro models and the in vivo systems they are intended to replace (in full or part). Therefore, the aim here was to match in vivo doses, expressed in terms of mass deposited per unit surface area, with the in vitro doses, and these agreed within approximately 10% (ca 10 ng/cm^2^ and 100 ng/cm^2^, low and high doses, respectively (Table 3)). In both models, there was no evidence of adverse effects based on molecular changes or other toxicity measures.

There are clearly uncertainties surrounding dose estimates, for example, in our study, the assumption of complete deposition for the in vitro system, when the actual delivered dose is likely to be lower as only a fraction of the administered mass will reach the cells at the bottom of the well over the exposure period [52,53]. There are also uncertainties associated with the estimation of doses in vivo, which rely on multi-parameter deposition models, although it is difficult to judge their significance. Another complication is that clearance mechanisms (e.g., mucociliary) are operational in vivo that are not present in vitro. It is also important to note that the in vitro system used here only reflects part of the respiratory system (conducting airways), which may also have implications for comparability. For example, Teeguarden et al. [31] exposed Balb/c mice to a high concentration aerosol (20 mg/m^3^) of superparamagnetic iron oxide NPs for 4 h and murine epithelial and macrophage cell types also for 4 h at the same dose level. They found good correspondence between target cell doses triggering inflammatory processes in vitro and in vivo in the alveolar macrophage population, but not in the epithelial cells of the alveolar region.

Aerosol mass concentration has traditionally been used as the primary dose metric for in vivo inhalation toxicology studies. However, it has been suggested that other metrics, in particular deposited doses of different forms, are more relevant for predicting the biological effects of particles, as they relate more specifically to the interactions between particles and respiratory tissues. For example, it has been proposed that deposited surface area is a more appropriate dose metric for acute inflammatory effects in the lungs from broadly spherical low-solubility particles [54,55]. Although there is currently no agreement on the most appropriate metric(s), overall, there is a growing consensus that determining and reporting a range of aerosol exposure and dose metrics (e.g., those related to mass, surface area, volume, and particle number (aerosol agglomerate and primary particle)) is important to allow appropriate interpretation and comparison of study results, both now and in the future [56]. We therefore measured and calculated values for a wide range of aerosol and deposited dose metrics (Tables S3 and S4). A number of other FeO_x_ inhalation studies have reported metrics beyond simply aerosol mass concentrations: Including, aerosol surface area concentration [57], deposited mass [31,32,57] and deposited surface area [32]. Unfortunately, it is difficult to make direct comparisons with these studies based on the additional metrics because of complicating factors, such as indications of lung overload [57], and the use of different animal models (mice, Pettibone et al. [32]; Teeguarden et al. [31], without additional data and analysis. Our results are, however, broadly consistent with those of a study comparing surface area doses (cm^2^/g-lung) of intratracheally instilled NPs with the resulting acute influx of polymorphonuclear (PMN) cells from a range of published studies. Schmid and Stöger [55] found significant PMN numbers (>30% of total BALF cells) only at surface area doses above 175 cm^2^/g-lung for a range of materials considered to be of low intrinsic toxicity, with very little response (<10% PMN) below around 20 cm^2^/g-lung. This is in line with our finding of no or limited PMN influx for our exposures, which resulted in maximum deposited surface area doses of approximately 10 cm^2^/g-lung. In a related study Hadrup and Saber [58] found that intratracheally instilled Fe_2_O_3_NPs also produced low levels of neutrophilia for similar surface area doses. These results are in contrast to findings for a number of other metal NPs (Co, Ni, Zn) for which significant PMN influx was seen at these low doses [55], which provides further support for our finding of the low toxicity of FeO_x_NPs.

The sizes of the primary particles used in our study were broadly similar to the environmental particles (Table 1), with the exception of the γ-Fe_2_O_3_, which were significant smaller (5.5 ± 2.1 nm vs. 32.3 ± 22.3 nm). The aerosol agglomerates (130–140 nm (CMD)) were at the lower end of the size distribution for the environmental agglomerates (typically few hundred nm), which would impact to some extent on the deposition pattern within the lung, although smaller iron-rich agglomerates have been identified in other studies. For example, particles with diameters between 5 and 80 nm were measured in urban Prague [59] and ca 50 nm in East Asia [60,61]. The form of the agglomerates may also differ. Although similar in appearance (Figure S2 and Figure 4), the degree and type of bonding between the primary particles may differ in the two cases due to the different processes under which they were formed. This may impact upon the surface area dose and the actual surface form may also impact on toxicity. The experimental particles clearly also lacked the trace elements of the environmental particles, which may also influence the effects of exposure. These factors are worthy of further investigation.

Measurements of ultrafine particles at roadsides in Birmingham and Newcastle found the iron content of PM_0.1_ in the range 10–100 ng/m^3^ [16]. These results are consistent with the findings of a review of atmospheric metal nanoparticles, which found that iron content ranged from 0.73 ng/m^3^ (rural Finland) to 186 ng/m^3^ (Los Angeles) [13]. These mass concentrations are significantly lower than those used in this study (50 and 500 µg/m^3^), however short-term exposures (3 h/d × 3 d = 9 h) were used here rather than the chronic exposures that occur in the general population. In terms of delivered dose, 50 µg/m^3^ for 9 h is broadly equivalent to 50 ng/m^3^ for a complete year and, whilst this ignores the influence of clearance processes, is thus broadly indicative of an annual exposure. The equivalent for the high concentration, 500 µg/m^3^, is 10 years exposure at 50 ng/m^3^. To put the concentrations in a wider context, average measured values of iron-rich dusts in roadside PM_10_ and PM_2.5_ in London and Birmingham were, respectively, 6.1 µg/m^3^ and 1.4 µg/m^3^ [62], also significantly lower than those in this study. However, the concentrations used here are consistent with the upper range of those found within the iron-rich environment of the London Underground (e.g., range 56 to 250 µg/m^3^ iron in PM_2.5_ on Hampstead station platform) [9].

## 4. Materials and Methods

### 4.1. Iron Oxide Nanoparticles

Dispersions in water of the following forms of iron oxide nanoparticles (FeO_x_NPs): α-Fe_2_O_3_, γ-Fe_2_O_3_ and Fe_3_O_4_, were obtained from Promethean Particles Ltd., Nottingham, UK. Separate batches were used for the in vitro and in vivo studies, and all products were provided at 10 mg/mL, except the Fe_3_O_4_ for the in vivo studies, which was 18 mg/mL. The primary particle sizes were intended to match those found in the environment. The individual suspensions were combined to produce a stock mixture (FeO_x_-mix) containing the different oxides in broadly the same (number-based) proportions as found in the environment [16], as described in Supplementary Information 2.

### 4.2. Characterisation of FeO_x_NPs in Dispersions

To characterise the particle size distribution and morphology of the iron oxide nanoparticles, very small droplets of the individual FeO_x_NP dispersions and the mixture were pipetted onto TEM grids and the excess moisture was allowed to dry. To measure the size of the primary particles, TEM imaging of samples was carried out using a FEI Tecnai F20 Transmission Electron Microscope working on high tension at 200 kV, with an extraction voltage of 4450 eV. Bright-field particle imaging in TEM mode was carried out using the Gatan Digital Micrograph attached to the instrument. For identifying likely particles, the instrument scanning TEM (STEM) mode was used in conjunction with high angle annular dark field (HAADF) detection. The Gatan Digital Micrograph software was used to measure the particle diameters of 40 NPs of each iron oxide. Additional high-resolution TEM images were taken with a JEOL 3000F Transmission Electron Microscope (JEOL Inc., Tokyo, Japan).

### 4.3. Cell Culture and FeO_x_NPs Preparation

Human bronchial epithelial cells (BEAS-2B) were grown in a complete medium consisting of RPMI 1640 GlutaMax supplemented with 10% FBS (Sigma-Aldrich, St. Louis, MO, USA) and 1% penicillin streptomycin (Gibco; Thermo Fisher Scientific, Loughborough, UK). Cells were maintained in a 5% CO_2_ incubator at 37 °C. Stock suspensions of FeO_x_NPs (10 mg/mL) prepared in sterile distilled water were sonicated for 1 min using an ultrasonic bath, then diluted to the required concentration using serum-containing media.

### 4.4. Cell Cytotoxicity Assays

Cells were plated in 96-well plates at 4 × 10^3^ cells per well in serum-containing media and cultured in a 5% CO_2_ incubator at 37 °C. After 24 h, cells were treated with different concentrations of the FeO_x_NPs or FeO_x_-mix (0.02, 0.1, 0.5, 2.5, 10, 50 and 200 μg/mL) at 37 °C for 24 h. CCK-8 cell viability assay was performed according to manufacturer’s protocol (Sigma-Aldrich). Cell viabilities in triplicate wells were measured as the absorbance (450 nm) of reduced WST-8 2-(2-methoxy-4-nitrophenyl)-3-(4-nitrophenyl)-5-(2,4-disulfophenyl)-2H-tetrazolium, monosodium salt) using an Infinite 200Pro microplate reader (Tecan Trading AG, Switzerland). Cytotoxicity was also assessed using the Lactate Dehydrogenase (LDH) Cytotoxicity Assay (Thermo Fisher Scientific, Loughborough, UK) according to the manufacturer’s protocol. Cells were prepared and treated with FeO_x_NPs as described above. FeO_x_NPs cytotoxic effects in triplicate wells were measured at 492 nm and 690 nm wavelengths, respectively, using a Multiskan Multisoft Primary EIA microplate photometer.

### 4.5. Cell Exposure Study for Transcriptomics and Metabolomics

Cells were plated in 6-well plates at 1.5 × 10^5^ cells per well in serum-containing media and cultured in a 5% CO_2_ incubator at 37 °C. After 24 h, cells were treated with FeO_x_-mix (0.003, 0.03, 0.3 μg/mL) or Fe_3_O_4_ (0.03 µg/mL) at 37 °C for 24 h. The concentration of 0.03 μg/mL was selected on the basis of the preliminary in vitro study results as approximately the maximum concentration before cytotoxicity was clearly evident and was chosen to avoid the detection of responses that were simply secondary to cytotoxicity and not due to the treatment of itself. For the FeO_x_-mix concentrations a factor of 10 below (0.003 μg/mL, no evident cytotoxicity) and 10 above (0.3 μg/mL, low cytotoxicity) were also used to investigate potential dose response. After treatment, cells were processed for RNA and metabolite extraction as described later. Methods for transcriptomic and metabolomic analyses are described later.

### 4.6. In Vivo Exposure of Sprague–Dawley Rats (Nose-Only Inhalation)

The experiments were performed within the legal framework of the United Kingdom under a project license granted by the Home Office of Her Majesty’s Government. All procedures involving the animals were performed in accordance with the Animals (Scientific Procedures) Act 1986 (licence number PPL 30/3071, approved on 23 May 2013 by UK Secretary of State). Male, pathogen-free. Sprague–Dawley (SD) rats (9–13 weeks) were purchased from Harlan, UK. Rats were randomly assigned into groups and exposed to aerosolized Fe_3_O_4_ nanoparticles, the FeO_x_-mix or milliQ water (controls) for 3 h per day for 3 days. For Fe_3_O_4_ two concentrations were delivered, whereas for the FeO_x_-mix, only the higher concentration was delivered. Target aerosol concentrations of 50 µg/m^3^ and 500 µg/m^3^, were chosen to broadly match the sub- and low- cytotoxicity doses administered in the in vitro study (0.03 and 0.3 μg/mL), as justified above and discussed further below. These concentrations are significantly in excess of reported environmental levels (e.g., Fe content of ultrafine fraction 10–100 ng/m^3^ [16] and of total PM 400–600 ng/m^3^ [8]). Following exposure, the rats were returned to their cages for a period of 1 d or 7 d.

### 4.7. Aerosol Exposure System

A schematic of the nose-only aerosol inhalation exposure system is shown in Figure S1. This system is similar to that used in our previous nose-only nanoparticle inhalation exposure studies (e.g., Guo et al. [63]). The aerosol was produced using a TSI constant output atomizer (Model 3076, TSI Inc., Shoreview, MN, USA) supplied with filtered compressed air at a constant pressure of 35 psig. After production, the aerosol was first passed through a diffusion dryer (Model DDU570, TOPAS GmbH, Dresden, Germany) before being diluted with a variable bridge diluter. The aerosol then entered a stainless-steel neutralizing and mixing chamber, where it was diluted with humidified filtered air. The relative humidity and temperature of the aerosol delivered to the exposure chambers were constantly measured and recorded using a Humidity and Temperature Meter, (model HMT330, Vaisala, Vantaa, Finland). Throughout all exposures, relative humidity was >50% and temperatures were between 20 and 22 °C. The aerosol then entered a custom-built nose-only exposure manifold (EMMS, Bordon, UK) [64].

### 4.8. Characterisation of FeO_x_ Nanoparticle Aerosols

The aerosol particle size distribution and number concentration were continuously measured from an animal port on the exposure manifold using a scanning mobility particle sizer (SMPS model 3936N76, TSI Inc., Shoreview, MN, USA) and condensation particle counter (CPC model 3775, TSI Inc., Shoreview, MN, USA). Average and real-time aerosol mass concentrations were determined gravimetrically and using a TEOM^TM^ ambient particulate monitor (Model 1400a, Thermo Scientific, Franklin, MA, USA), respectively, from a sampling spur installed just before the exposure manifold. Gravimetric samples were taken onto 47 mm EMfab™ Pallflex^®^ filters (TX40HI20WW, PALL Life Sciences, Portsmouth, UK) held in an in-line stainless steel filter holder (PALL Life Sciences, Portsmouth, UK) using a GilAir™ Plus Sampling Pump (Sensidyne, FL, USA) at 2 lpm. The morphology of the aerosol particles delivered to the exposure manifold was determined with high-resolution transmission electron microscopy (TEM) (JEOL 3000F, JEOL Inc., Tokyo, Japan). Samples for TEM were taken directly onto 400 mesh copper TEM grids with lacey carbon film using a mini particle sampler (MPS, Ecomeasure, Saclay, France) at a flow rate of 0.3 lpm for 3 min. For each sample, projected area equivalent diameters were calculated for 140 randomly selected particles using the image analysis software ImageJ v1.52a). Real-time concentration information was used to maintain a constant aerosol concentration throughout exposure using the bridge diluter installed in the system. From the aerosol measurements the following additional aerosol metrics (per m^3^) were estimated: Number of primary particles; particle volume; and particle surface area. Further details are given in Supplementary Information 2.

### 4.9. Dose Estimation

For the in vitro exposures, assuming all particles in solution deposit onto the cells over the 24-h exposure period, the mass dose per unit area, *DA*_in vitro_, (µg/cm^2^), can be calculated as:

$$DA_{\mathrm{in}\;\mathrm{vitro}}=\frac{CV_{admin}}{A_{well}}$$

where *C*, is the nanoparticle solution concentration (µg/mL), *V_admin_* (mL), is the volume of nanoparticle solution and, *A_well_*, is the surface area of the in vitro well (cm^2^) and cells exposed. Deposited doses in vivo, *D*_in vivo_ (µg), were determined using the following formula:

$$D\mathrm{in}\;\mathrm{vivo}=C\cdot MV\cdot T\cdot DE\times 10^{-6}$$

where *C* (µg/m^3^) is the aerosol mass concentration, *MV* (mL/min), the rat minute ventilation, *T* (min), the exposure duration, and, *DE*, the deposition efficiency. Breathing parameters were measured using head-out plethysmographs (EMMS, Bordon, UK) for 5 male SD rats during 3-h exposures on different days. The average (±standard deviation) minute ventilation rate of 11 measurements was 205 ± 29 mL/min, in line with typical values for animals of similar mass found in the literature [65,66,67,68]. Particle deposition efficiencies in the rat lung were determined using the Multiple Path Particle Deposition (MPPD) model (version 3.04, Applied Research Associates, Inc., Albuquerque, NM, USA). Table S2 summarises the parameter values used in the simulations. Deposition efficiencies for the total lung, tracheobronchial region and alveolar region were 0.149, 0.046 and 0.103, respectively. Equivalent formulae were used to determine the deposited doses expressed in terms of particle number, volume and surface area (see Supplementary Information 2 for details).

To deliver broadly equivalent in vivo doses per unit area to the tracheobronchial region to the sub- and low- cytotoxic doses used in vitro, an equivalent aerosol concentration, *C_eq_* (µg/m^3^), was determined from the calculated in vitro dose per unit area, *DA*_in vitro_ (µg/cm^2^), using the following equation, derived from the equations above,

$$C_{eq}=\frac{A_{TB}\cdot DA_{\mathrm{in}\;\mathrm{vitro}}}{MV\cdot T\cdot DE}\times 10^{6}$$

where *A_TB_* (cm^2^), is the area of the rat tracheobronchial region, taken here as 22.5 cm^2^ [69].

### 4.10. Lung Tissue Samples and Bronchoalveolar Lavage (BAL)

Rats were sacrificed by exsanguination by cardiac puncture under isoflurane anaesthesia (induced at 5%, maintained at 1.5–2% in 100% oxygen). Bronchoalveolar lavage fluid (BALF) was collected via tracheal cannula with 2 × 7 mL aliquots of phosphate buffered saline. BALF was centrifuged at 1500× *g* for 10 min. Cells from both aliquots were pooled for analysis of total and differential cells. The BALF supernatant from the first wash was retained for gross toxicity analysis. The apical and azygous lung lobes were tied off and snap frozen in liquid nitrogen for transcriptomics and metabolomics analyses.

### 4.11. BALF Analysis

BALF cells were reconstituted to 1 × 10^6^ cells/mL and 100 μL was spun onto slides using a cyto-centrifuge. Staining for differential cell count was performed using the Shandon Kwik-Diff Stains kit (Thermo Scientific, Loughbrough, UK). At least 300 cells on the slides in total were counted and identified as macrophages, neutrophils, eosinophils, basophils and lymphocytes according to standard morphology under a microscope with 400× magnification. The lactate dehydrogenase (LDH) (Promega) assay was used to assess general cytotoxicity using the supernatant of the first BAL wash (samples were stored at −80 °C before analysis). Assays were performed in black-out 96-well plates and 50 µL of BALF was added to 50 µL of reconstituted substrate solution and incubated for 30 min at room temperature in the dark. Then 50 µL of stop solution was added to each well and the absorbance (Ab) was measured at 492 nm.

### 4.12. Metabolite and RNA Extraction from BEAS-2B Cells and Lung Tissues

BEAS-2B cells were washed rapidly with PBS, twice, before the well plates were quenched on liquid nitrogen for 60 s. Next, 80% methanol (pre-cooled on dry ice) was added to each sample and the cells were scraped from the bottom of each well and transferred to glass vials. The cell lysates were subjected to RNA or metabolite extraction, and subsequent analyses. Total RNA was isolated using Qiagen’s mini RNeasy Kit and QIAshredder (Qiagen, Crawley, UK) according to the manufacturer’s protocol. RNA was quantified with a NanoDrop 1000 spectrophotometer (Thermo Scientific, Waltham, MA, USA), and the integrity of RNA verified with a 2100 Bioanalyzer (Agilent Technologies, Santa Clara, CA, USA). Intracellular metabolites were extracted using organic solvents (methanol: chloroform: water, *v*/*v*/*v* 2:2:1.8) [70]. After vortexing (30 s, three times, at 30 s intervals), incubation on dry ice (10 min) and centrifugation (10 min, 1800 rcf, −9 °C), the polar and non-polar solvent phases were collected and dried in a speed vac concentrator (Thermo Savant, Holbrook, NY, USA) for 4 hrs. All dried samples were then stored at −80 °C until metabolomics analysis.

Frozen lung tissues were homogenised in 8 mL/g (*v*/*w* wet mass) methanol and 2.5 mL/g (*v*/*w*) water using a bead-based homogenizer (Precellys 24; Stretton Scientific, Stretton, UK). Lung homogenates were split for both RNA and metabolite extractions, following the same procedures as described above.

### 4.13. Microarray-Based Gene Expression Profiling of BEAS-2B Cells and Lung Tissue Extracts

The procedures for microarray analysis were performed according to the manufacturer’s protocols (Agilent Technologies, Santa Clara, CA, USA). Briefly, after the synthesis of cRNA from 50 ng of total RNA per sample, the probe RNA was amplified and labelled with Cy3-CTP (Agilent One-Color RNA Spike-In Kit and QuickAmp Labelling kit; Agilent Technologies). The Cy3-labelled cRNA was then fragmented and hybridized to Agilent’s SurePrint G3 Rat Gene Expression v2 8 × 60 k microarrays (Design ID: 074036), at 65 °C for 17 h. The hybridised microarrays were scanned with aG2565BA microarray scanner (Agilent Technologies) at A_535_ for Cy3. Microarray data were background-corrected, quantile-normalised between arrays and analysed using Qlucore Omics Explorer software (Qlucore, Lund, Sweden) to examine the effect of treatment on the transcriptional response. Samples that did not pass all 10 Evaluation Metrics in the QC (Quality Control) report were excluded in the analysis. Batch effects linked to the time of data acquisition were corrected on Qlucore Omics Explorer by setting up a General Linear Model (GLM) to eliminate the factor of batch effects. For microarray analysis on BEAS-2B cells, 34758 variables were identified after variance filtering for analysis. Effects of different FeO_x_ (Fe_3_O_4_ and FeO_x_-mix at the same exposure concentration) or concentration (different concentrations of FeO_x_-mix) were analysed separately further. For microarray analysis on rat lung tissues, 27104 variables were identified after variance filtering for analysis. Principal component analysis (PCA) was conducted to reduce the dimensionality of the data and to visually examine the similarities and/or differences in gene expression between treatment groups.

### 4.14. Metabolomics of BEAS-2B Cells and Lung Tissue Extracts

Direct infusion mass spectrometry (DIMS)-based polar metabolomics and lipidomics were performed as previously reported [71]. Dried polar and non-polar extracts were resuspended in methanol:water and methanol:chloroform solvents, respectively, with modifiers to aid electrospray ionisation, and then vortexed and centrifuged prior to analysis. Two quality control (QC) samples were prepared, one each for the BEAS-2B and lung tissue studies, by pooling an aliquot of each biological sample from the respective studies. Mass spectrometry analyses of the biological and QC samples were conducted using a high-resolution Fourier transform mass spectrometer (Orbitrap Elite, Thermo Fisher Scientific, Bremen, Germany) equipped with a Triversa chip-based nano-electrospray ion source (Advion Biosciences, NY, USA) using conditions as described previously [71]. Mass spectra were collected using a selected-ion-monitoring (SIM) stitching method from *m/z* (mass-to-charge ratio) 50–620 for polar metabolites and *m/z* 190–1200 for lipids, and then processed, normalised, missing value-imputed and generalised log-transformed, all as reported previously [71]. Univariate statistical analysis and PCA were conducted to examine the effect of treatment on the metabolic and lipidomic responses.

## 5. Conclusions

This study, the first of its kind to use FeO_x_NPs broadly matching the characteristics of environmentally sampled high iron content nano-sized particles, both individually and in an environmentally representative mixture, found they had cytotoxic potential, but demonstrated a lack of pulmonary toxicity in an in vivo model at aerosol concentrations exceeding those found in the environment and a parallel in vitro study using matched exposure levels. Given the significance of iron in ambient air pollution, especially from brake wear [35] and within underground rail networks [9], this is positive news. The results contrast with those of similar in vivo inhalation studies using concentrated levels (x 10) of ambient air pollution particles, which demonstrate clear indications of pulmonary inflammation, including gene expression changes and increases in BALF immune cell influx [72,73], suggesting that the FeO_x_NPs within ambient air pollution do not make a significant contribution to the overall effects of ambient particulate matter on the respiratory system. However, the focus of this study was limited to acute effects on the lung. The lack of any significant changes in gene expression and no or limited effects on metabolites suggests that chronic pulmonary effects at the exposure levels used are unlikely, however this needs to be confirmed by future longer-term studies. Potential effects on other systems, including cardiovascular and central nervous, also need to be addressed in future studies. Clinical findings from the established use of iron oxide nanoparticles within a medical context may ultimately provide useful direct human risk data in this area, however the particles used may differ significantly from those identified in the ambient atmosphere (e.g., the use of biocompatible coatings) and so care must be taken in their extrapolation to an environmental context [44,74]. It is also important to recognise that the study used pure FeO_x_NPs, whereas in the environment, iron-rich particles typically contain low concentrations of other metals. Iron-rich particles may perhaps play a role in delivering other more toxic metals to lung tissues. Recent studies have, for example, suggested that despite the high concentrations of iron it is other metals, including copper and vanadium [75,76], that may be driving toxicity from brake dust particles. Future studies using similar FeO_x_NPs with typical metallic impurities are therefore needed to further explore the health implications of the inhalation of environmental nano-sized iron-rich particles.

## Figures and Tables

**Table 1:** Comparison of the FeO_x_ particles used in this study to the atmospheric measurements.

Characteristic	Iron Oxide		
	Fe_3_O_4_	α-Fe_2_O_3_	γ-Fe_2_O_3_
Primary particle size (TEM) (nm) Mean ± SD (sample size)	19.42 ± 16.37 (n = 40)	10.61 ± 8.82 (n = 40)	5.47 ± 2.15 (n = 40)
Size (nm) of primary spherules in High-Fe airborne nanoparticle clusters from environmental samples ^(a)^ Mean ± SD (sample size)	26.97 ± 20.88 (n = 8)	13.14 ± 11.61 (n = 5)	32.28 ± 22.34 (n = 11)

^(a)^ [16].

**Table 2:** Measured aerosol characteristics.

Aerosol Parameter	Fe_3_O_4_ Low Dose	Fe_3_O_4_ High Dose	FeO_x_-mix
Count Median Diameter (SMPS) (nm)	130.5 ± 2.0	143.3 ± 1.7	139.2 ± 3.3
Geometric Standard Deviation	1.54 ± 0.02	1.60 ± 0.01	1.60 ± 0.02
Number Concentration (particles/cm^3^)	7.15 ± 0.95 × 10^3^	7.16 ± 0.74 × 10^4^	7.48 ± 1.56 × 10^4^
Mass Concentration (µg/m^3^)	47.6 ± 3.5	487.4 ± 3.7	507.8 ± 4.0

**Table 3:** Deposited doses for in vivo study compared with in vitro.

Group	Treatment Conc. (µg/mL)	Aerosol Conc. (µg/m^3^)	Deposited Dose Lung (µg)	Deposited Dose Alveolar (µg)	Deposited Dose Tracheobronchial (µg)	Dose Per Unit Area ^†^ (ng/cm^2^)
In vitro						
FeO_x_-mix A	0.003	-	-	-	-	0.99
FeO_x_-mix B	0.03	-	-	-	-	9.9
FeO_x_-mix C	0.3	-	-	-	-	99
Fe_3_O_4_	0.03	-	-	-	-	9.9
In vivo						
FeO_x_-mix High Dose	-	508	8.4	5.8	2.6	110
Fe_3_O_4_ High Dose	-	487	8.0	5.6	2.5	110
Fe_3_O_4_ Low Dose	-	48	0.8	0.6	0.24	11

**^†^** Mass dose per unit cell culture surface area for in vitro study and per unit tracheobronchial surface area for in vivo.

## Data Availability

The data presented in this study are available from the authors upon reasonable request.

## Supplementary Materials

The following are available online at https://www.mdpi.com/1422-0067/22/2/556/s1: 1. Supplementary Information_1 HRV study.docx. 2. Supplementary Information_2 FeO_x_ mixture ratio calculations and additional dose metrics.docx. 3. Supplementary Figures including Figure S1 Schematic diagram of the experimental set-up used in the nose-only inhalation exposure study; Figure S2 Representative TEM images of the (a,b) α-Fe_2_O_3_; (c,d) γ-Fe_2_O_3_; and (e) Fe_3_O_4_ aqueous nanoparticle suspensions as provided by the supplier, and (f) the mixed iron oxide nanoparticle suspension; Figure S3 Principal component analysis (PCA) score plots of the gene expression (A, B) and metabolomics profiles (C&D in positive ion mode, and E&F in negative ion mode) of BEAS-2B cells treated with H2O and FeO_x_NPs. (A, C, E) Comparison at same concentration of FeO_x_-mix NPs and Fe_3_O_4_ NPs; Figure S4 Principal component analysis (PCA) score plots of the transcriptomic (A&B) and metabolomics (C&D) profiles of lung tissue from rats exposed to H_2_O and FeO_x_NP aerosols at 1 day (A&C) and 7 days (B&D) post-exposure. 4. Supplementary Tables including Table S1 FeO_x_NP inhalation studies; Table S2 MPPD model input parameter values for deposition efficiency determination; Table S3 Additional aerosol characteristics; Table S4 Additional dose metrics for in vivo inhalation studies.
