# Supplementary material for: Environmentally Relevant Iron Oxide Nanoparticles Produce Limited Acute Pulmonary Effects in Rats at Realistic Exposure Levels
Source: Int J Mol Sci. 2021 Jan 8;22(2):556. doi: 10.3390/ijms22020556 (PMC7827273; doi:10.3390/ijms22020556)
Supplement: Supplementary file 1 [file ijms-22-00556-s001.zip › Supplementary Figures.docx]

**Environmentally relevant iron oxide nanoparticles produce limited acute pulmonary effects in rats at realistic exposure levels**

Chang Guo, Ralf J. M. Weber, Alison Buckley, Julie Mazzolini, Sarah Robertson, Juana Maria Delgado-Saborit, Joshua Z. Rappoport, James Warren, Alan Hodgson, Paul Sanderson, James Kevin Chipman, Mark R. Viant, Rachel Smith

**Supplementary Figures**


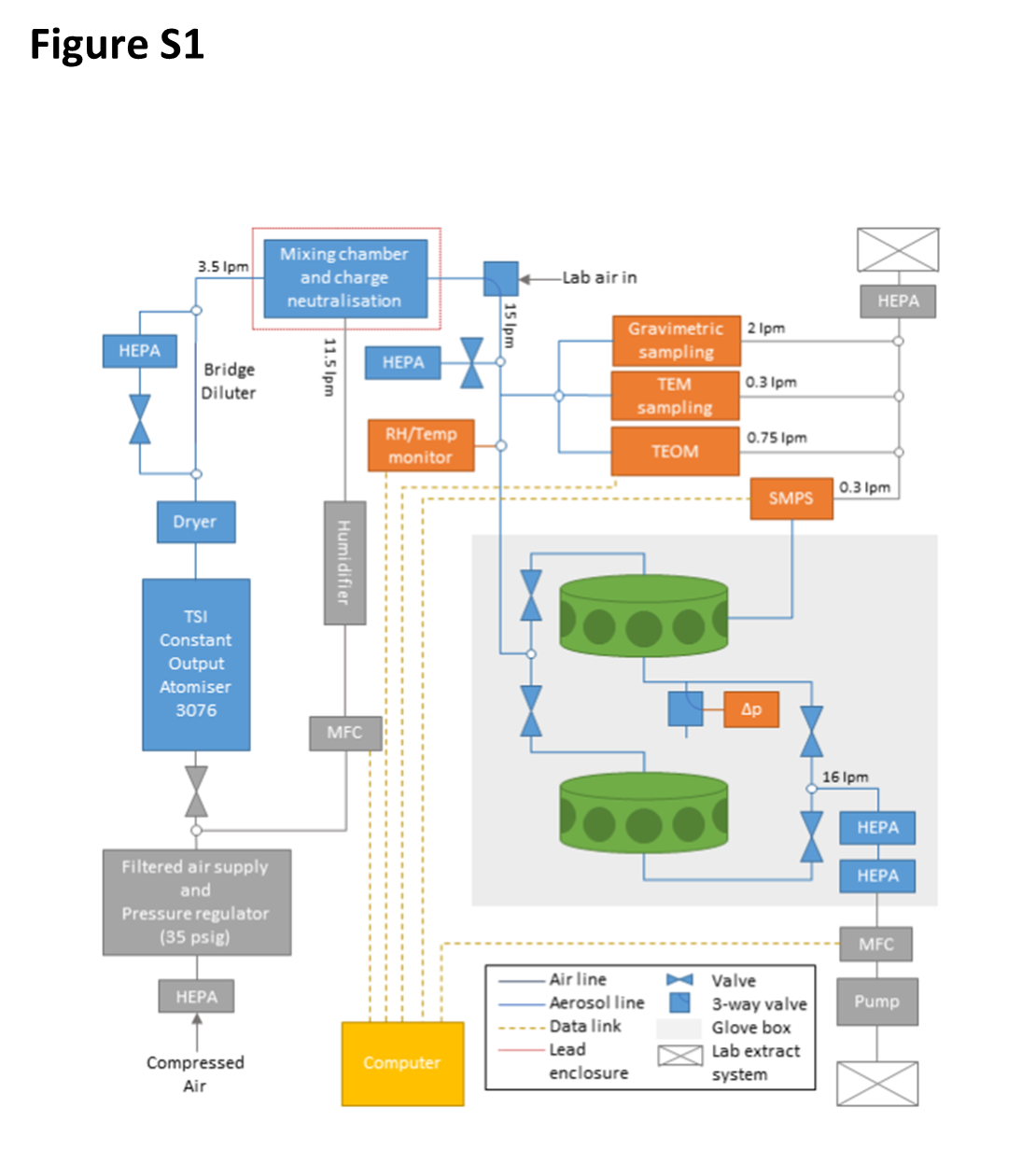


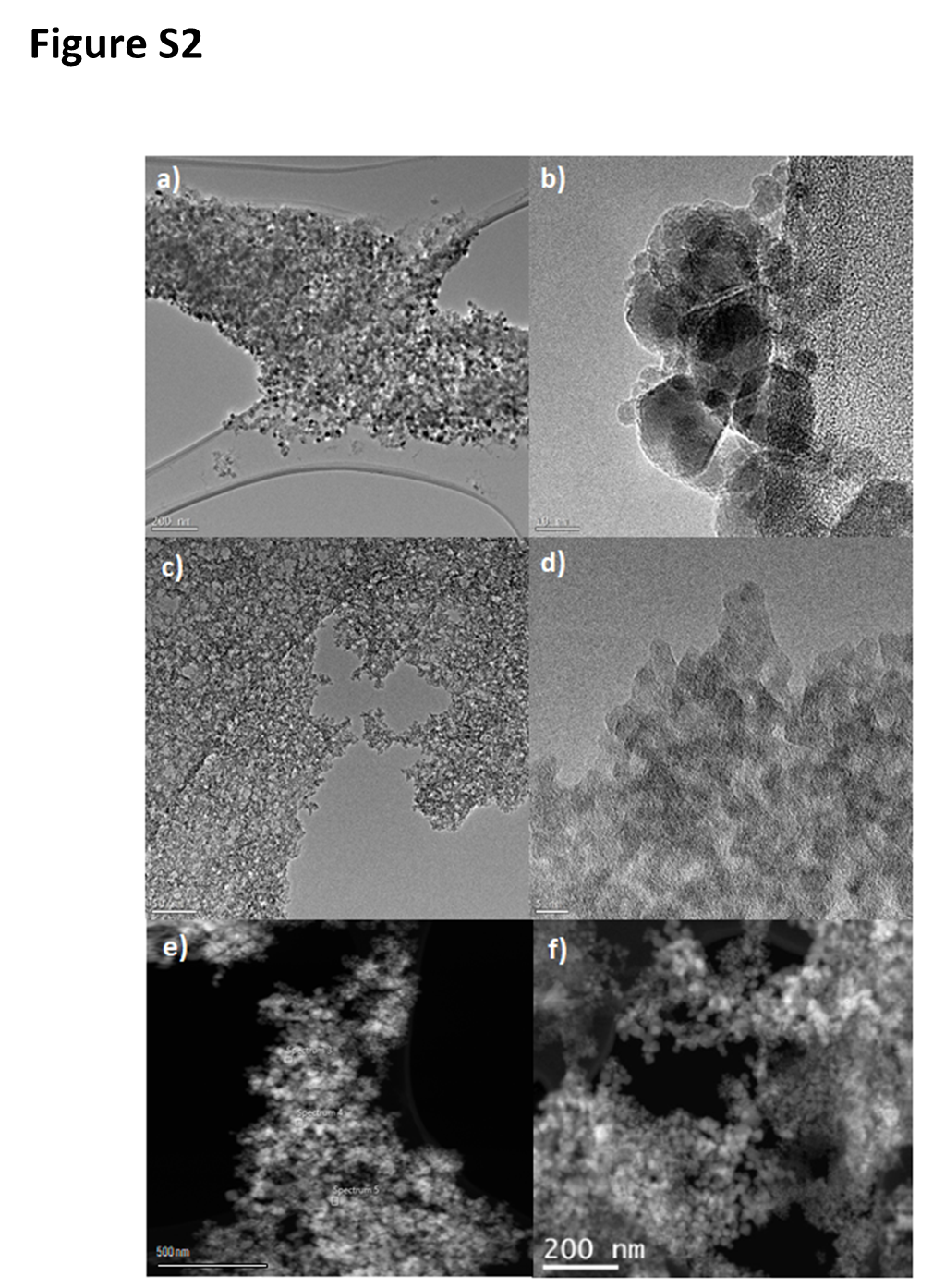


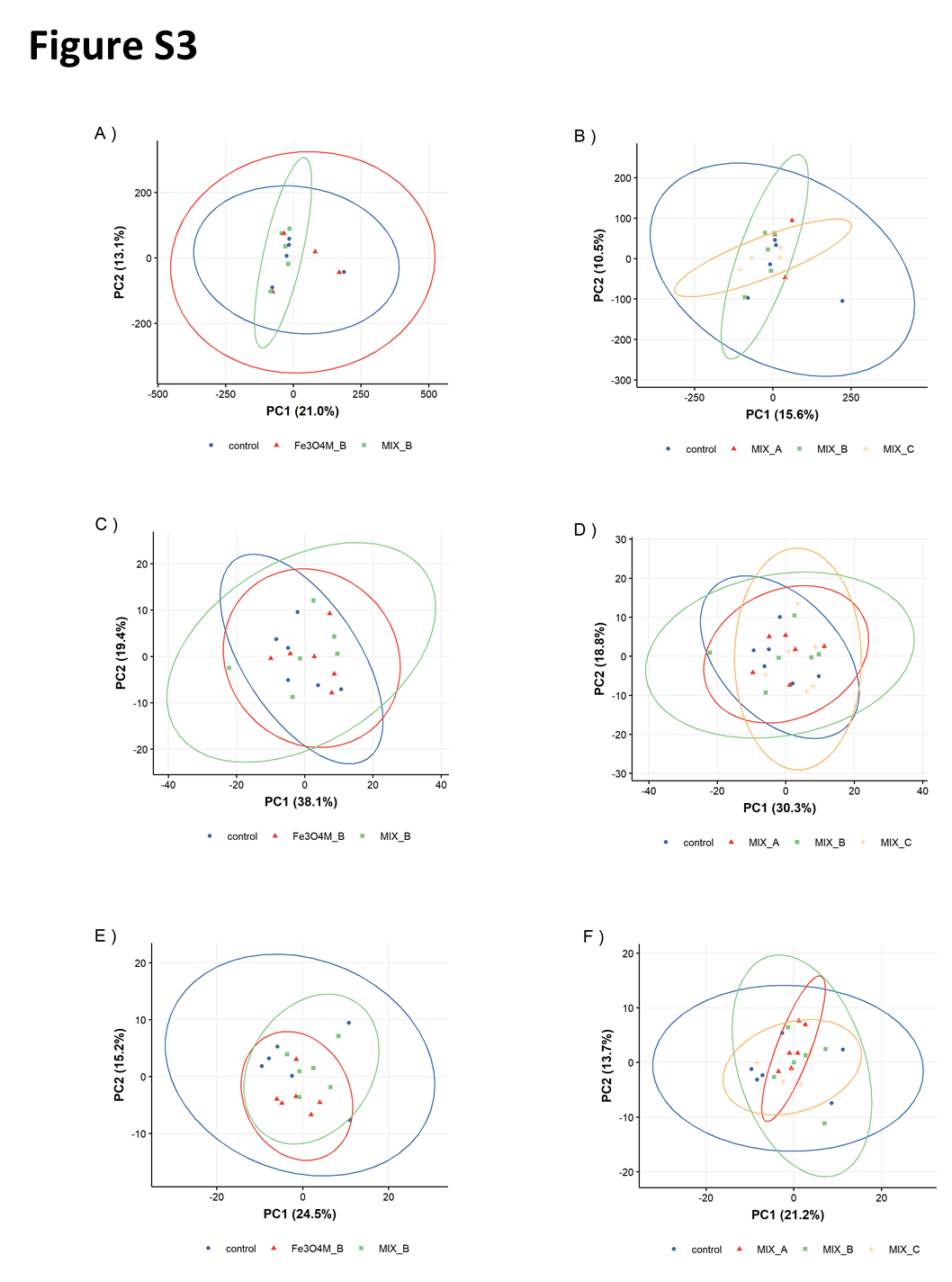


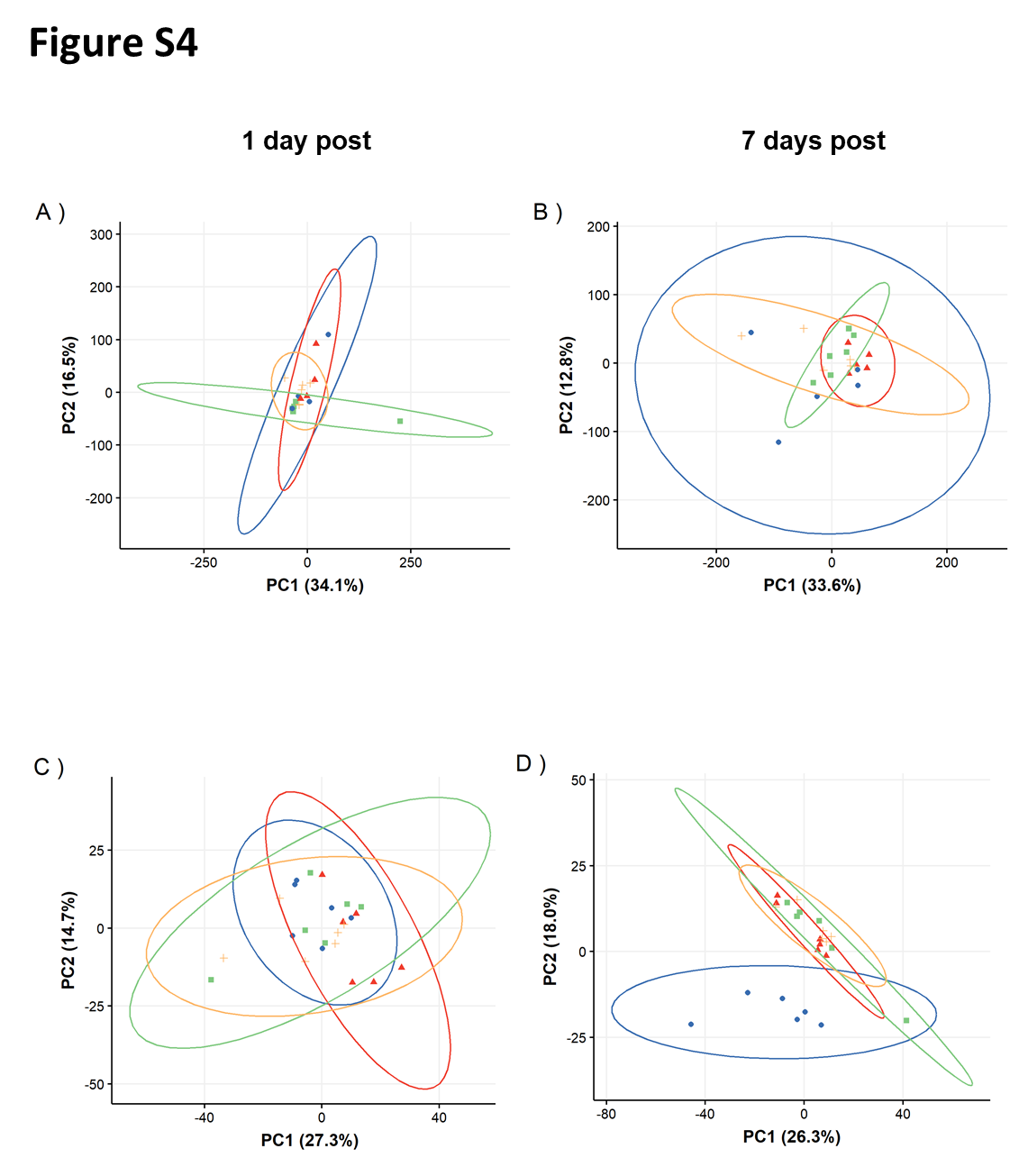


**Figure S1** Schematic diagram of the experimental set-up used in the nose-only inhalation exposure study. MFC: mass flow controller, HEPA: high-efficiency particulate air filter, SMPS: scanning mobility particle sizer, TEOM: tapered element oscillating microbalance.

**Figure S2** Representative TEM images of the a-b) α-Fe_2_O_3_; c-d) γ-Fe_2_O_3_; and e) Fe_3_O_4_ aqueous nanoparticle suspensions as provided by the supplier, and f) the mixed iron oxide nanoparticle suspension.

**Figure S3** Principal component analysis (PCA) score plots of the gene expression (**A-B**) and metabolomics profiles (**C&D** in positive ion mode, and **E&F** in negative ion mode) of BEAS-2B cells treated with H_2_O and FeOxNPs. (**A, C, E**) Comparison at same concentration of FeOx-mix NPs and Fe_3_O_4_ NPs. Symbols in the PCA plots indicate individual repeats of BEA-2B cells of control group (circle
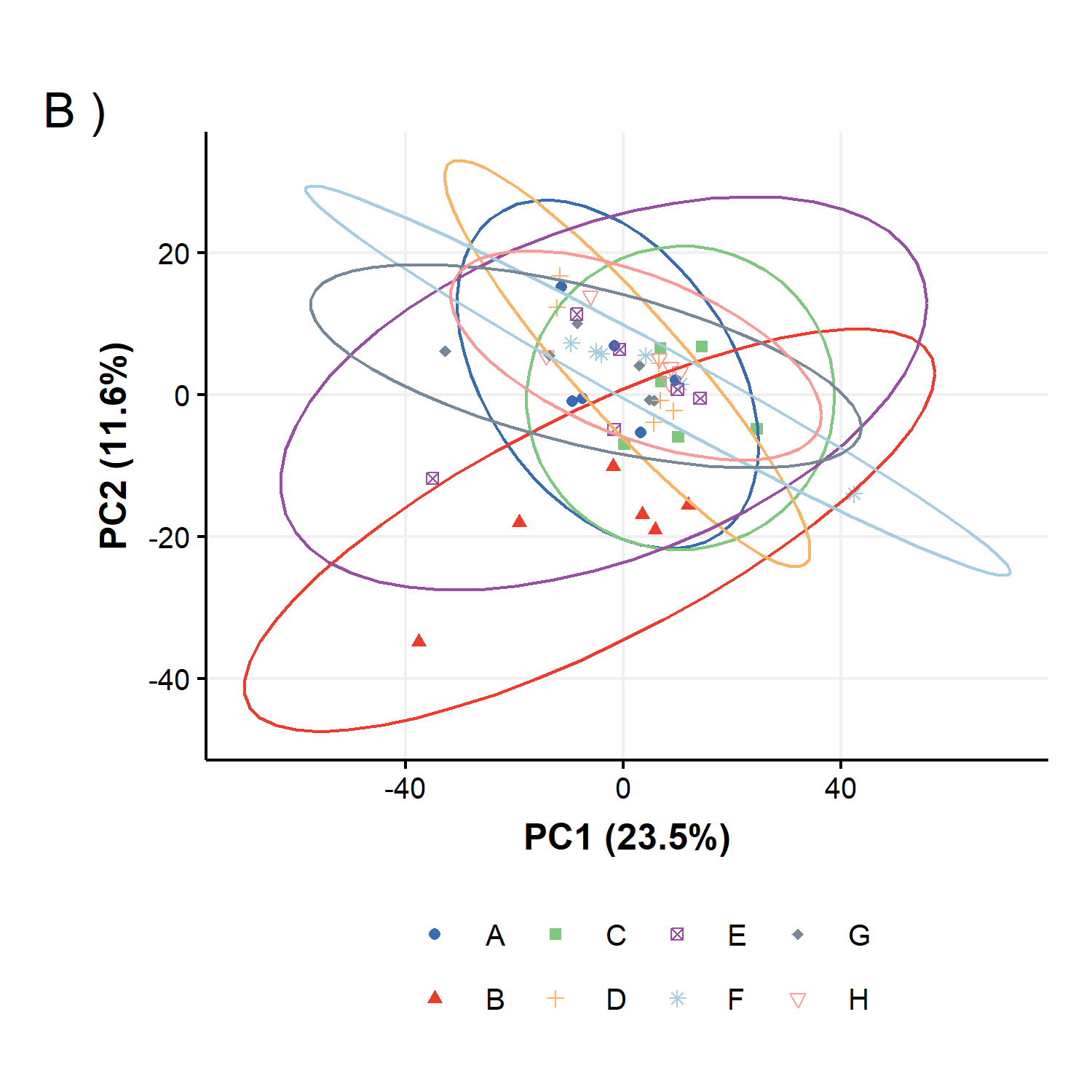
) or exposed to Fe_3_O_4_ B (triangle
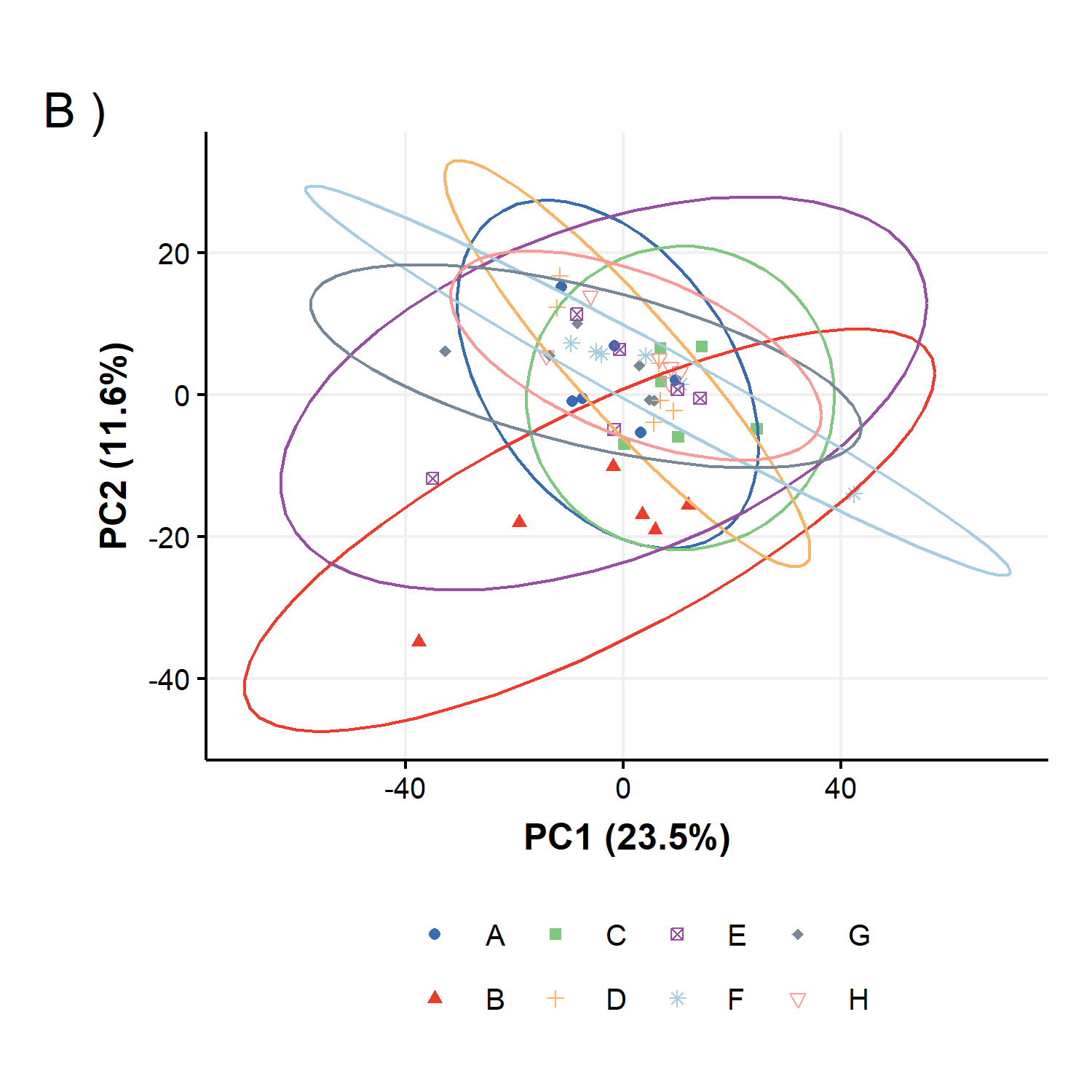
), or FeOx-mix B (square
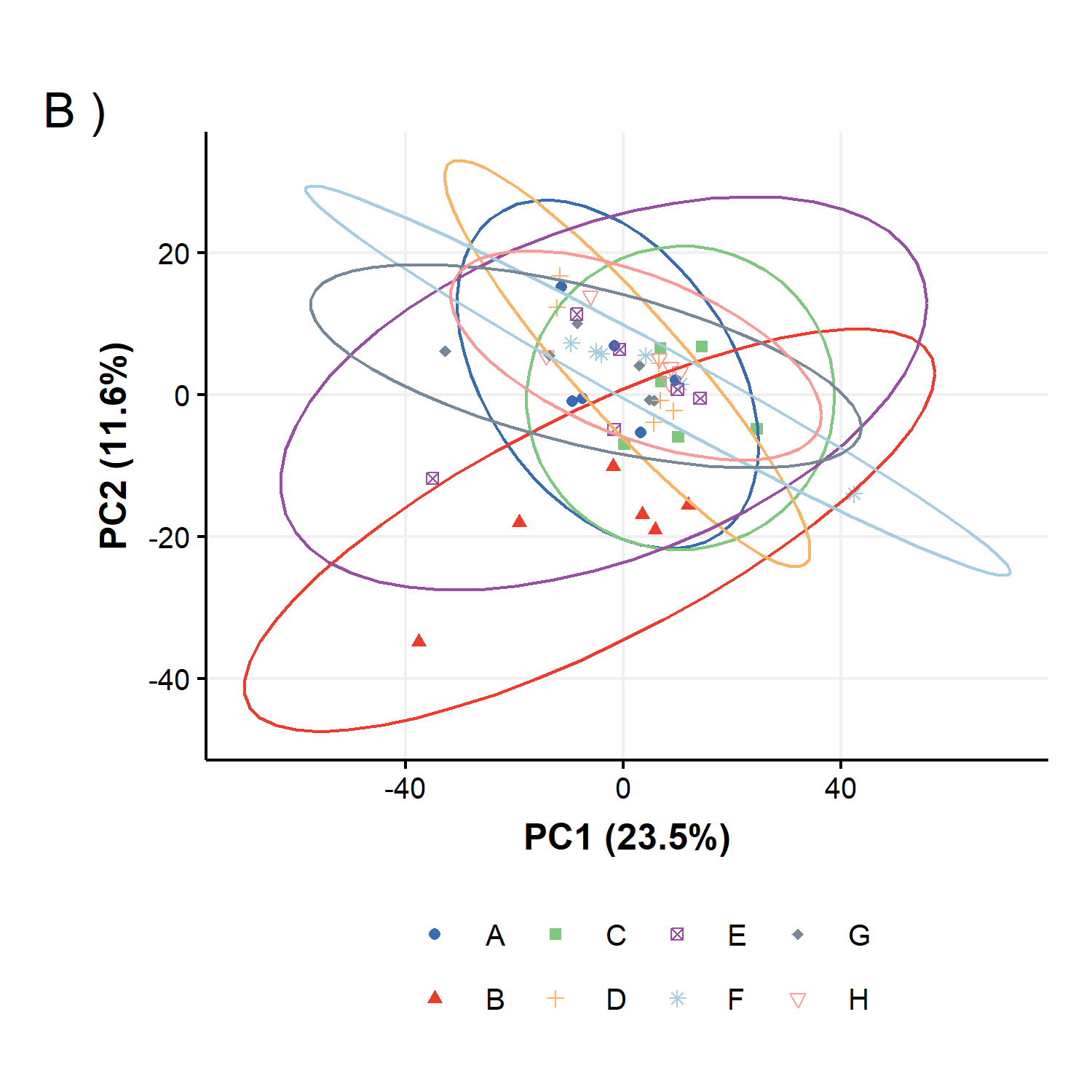
). (**B, D, F**) Comparison at different concentrations of FeOx-mix NPs. Symbols in the PCA plots indicate individual repeats of BEA-2B cells of control group (circle
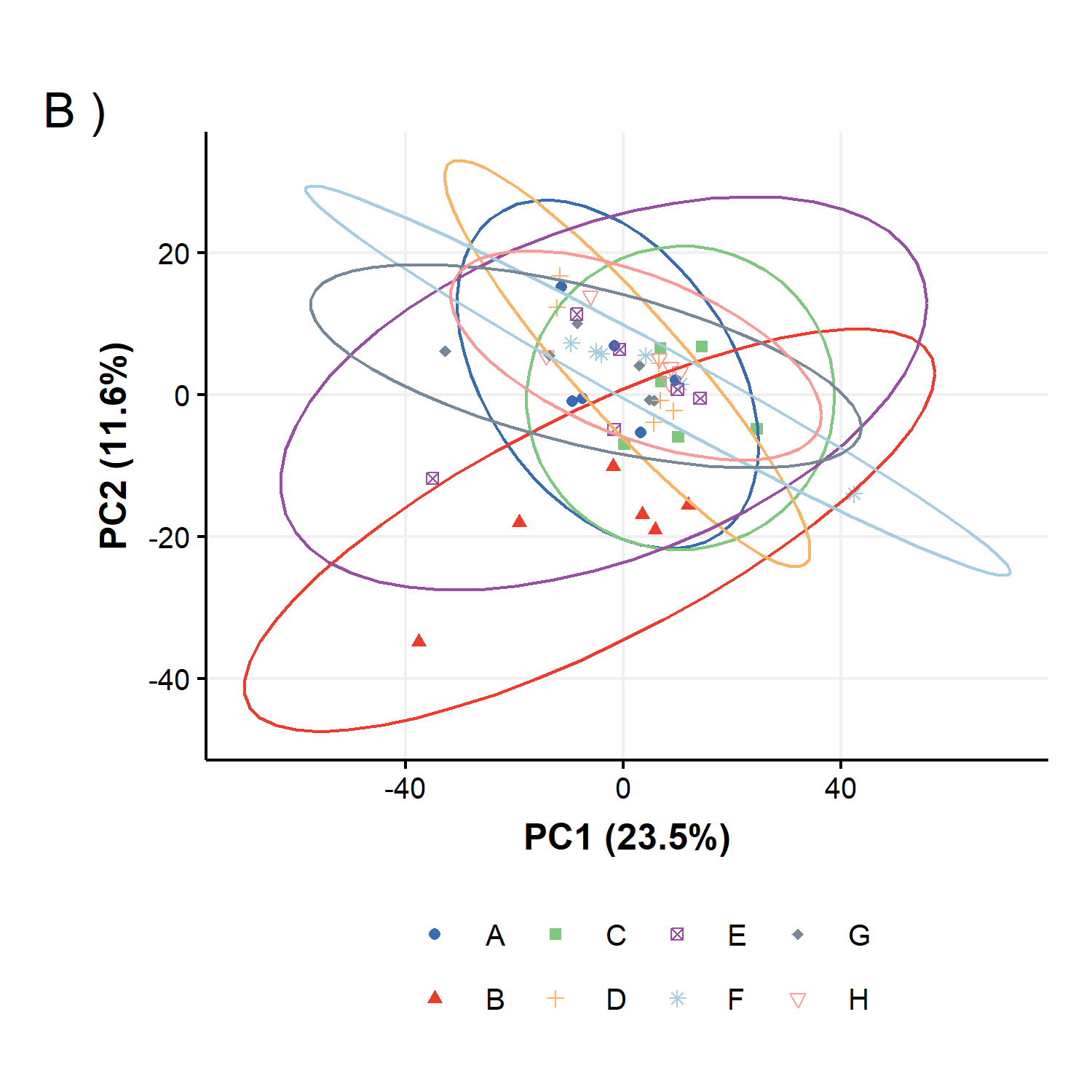
) or exposed to FeOx-mix A (triangle
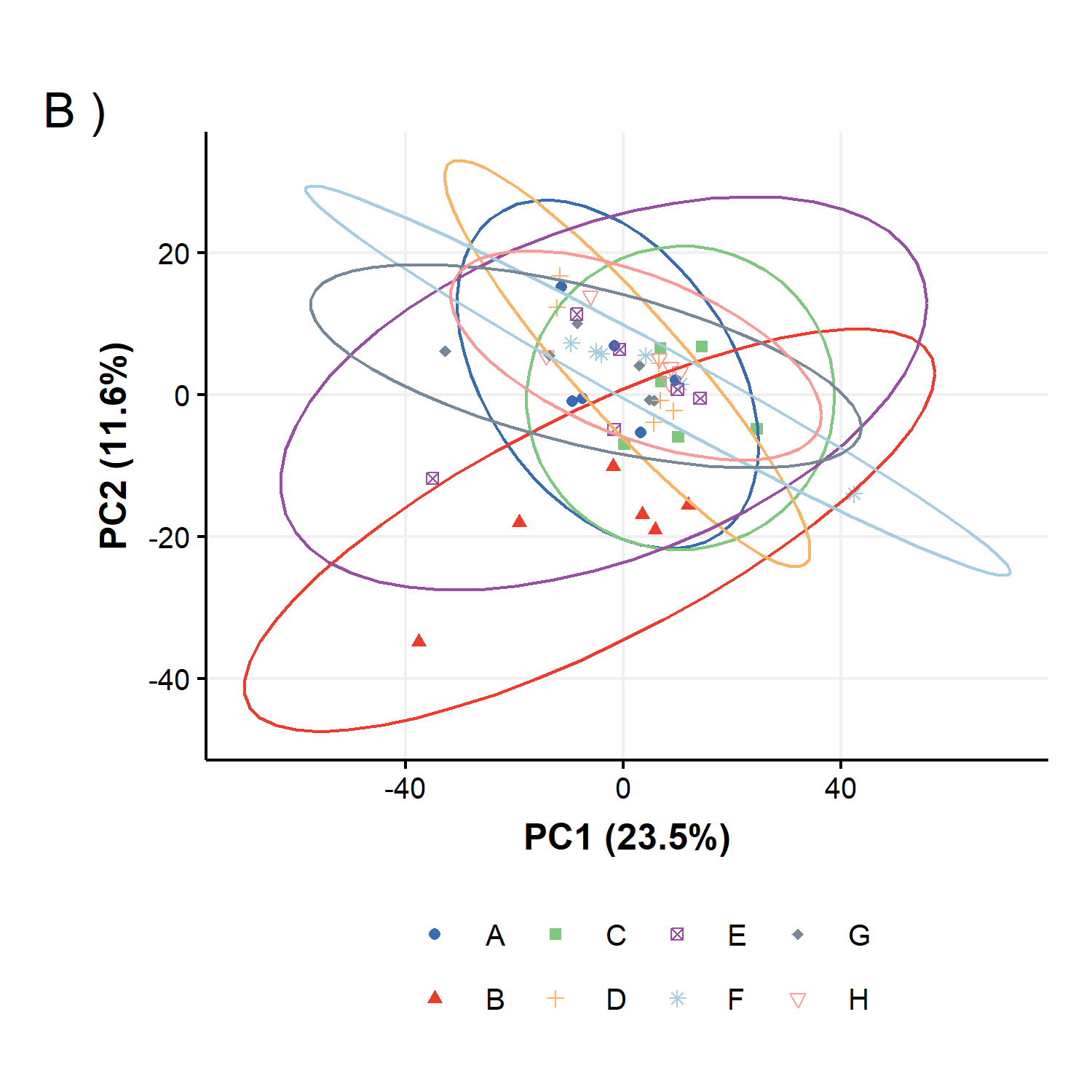
), FeOx-mix B (square
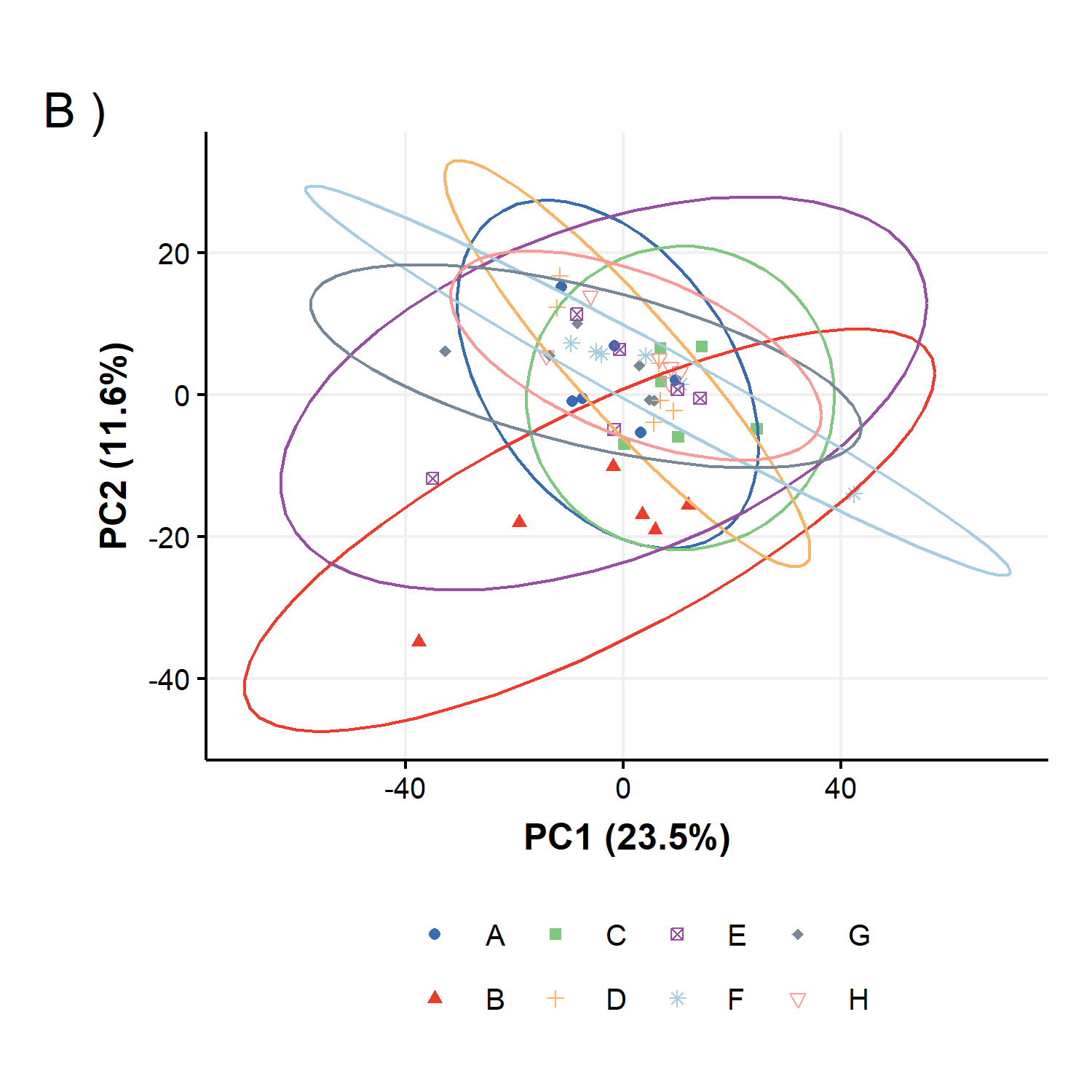
), and FeOx-mix C (plus
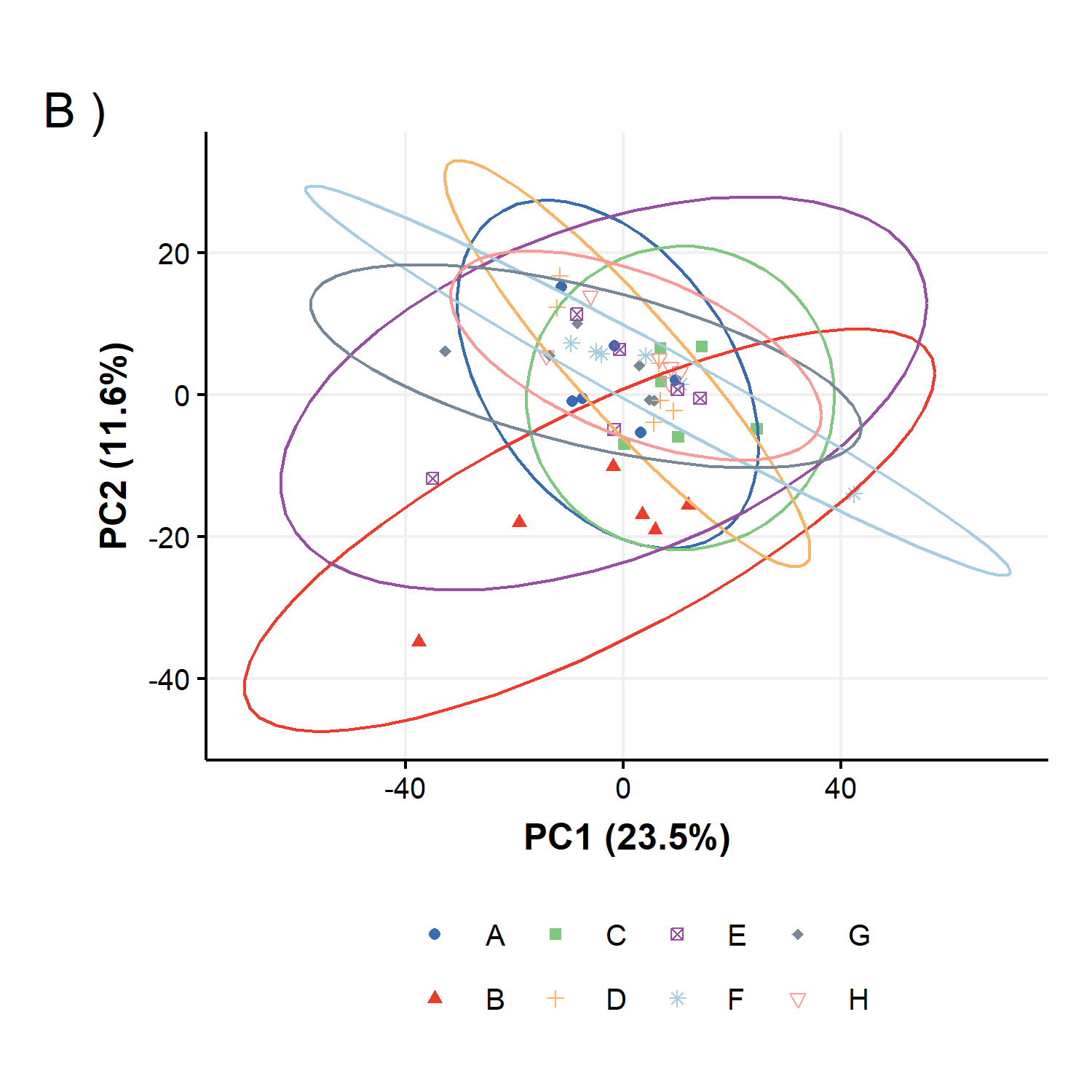
).

**Figure S4** Principal component analysis (PCA) score plots of the transcriptomic (**A&B**) and metabolomics (**C&D**) profiles of lung tissue from rats exposed to H_2_O and FeOxNP aerosols at 1 day (**A&C**) and 7 days (**B&D**) post-exposure. Symbols in the PCA plots indicate individual samples of rat lungs exposed to Water (circle
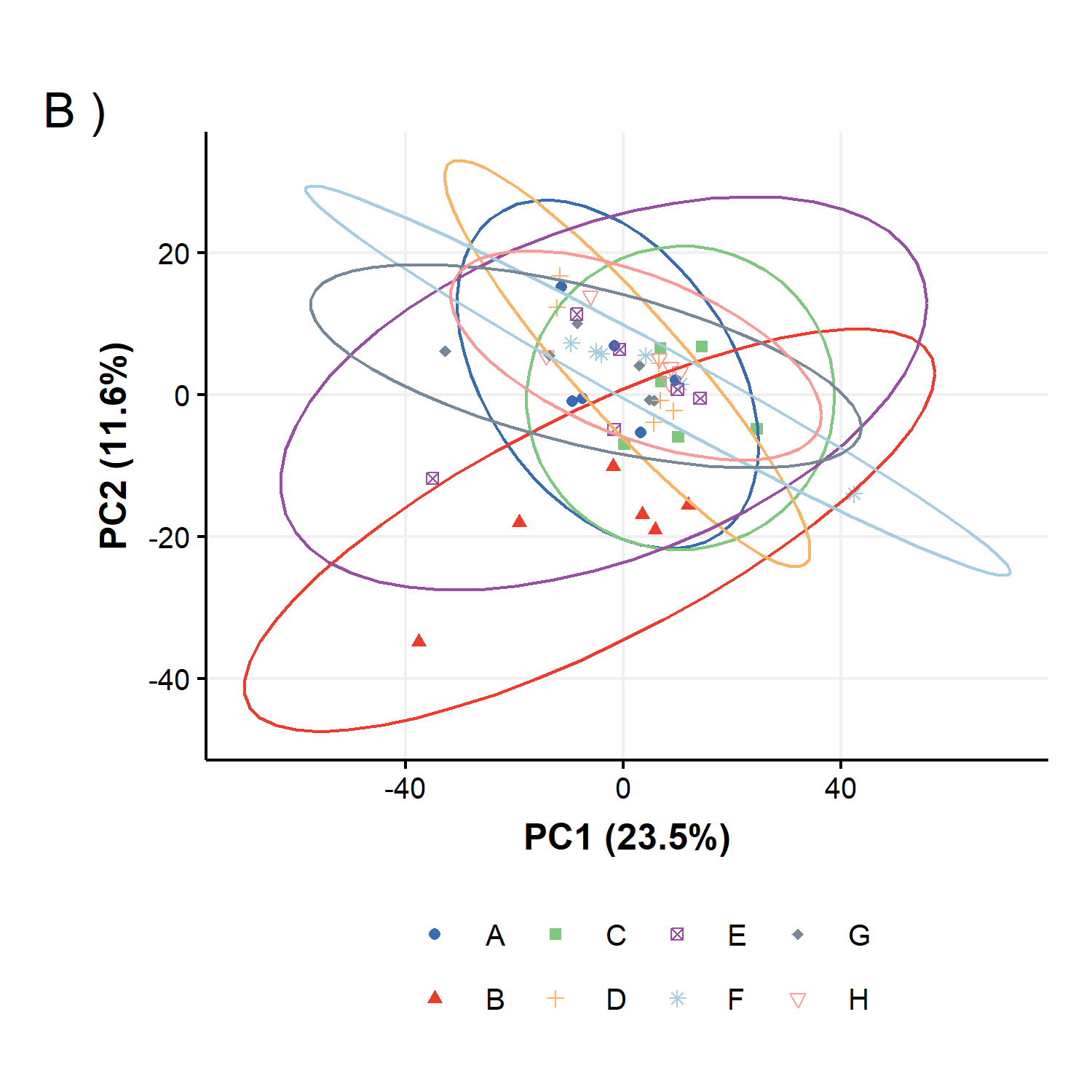
), Fe_3_O_4_ (low dose) (triangle
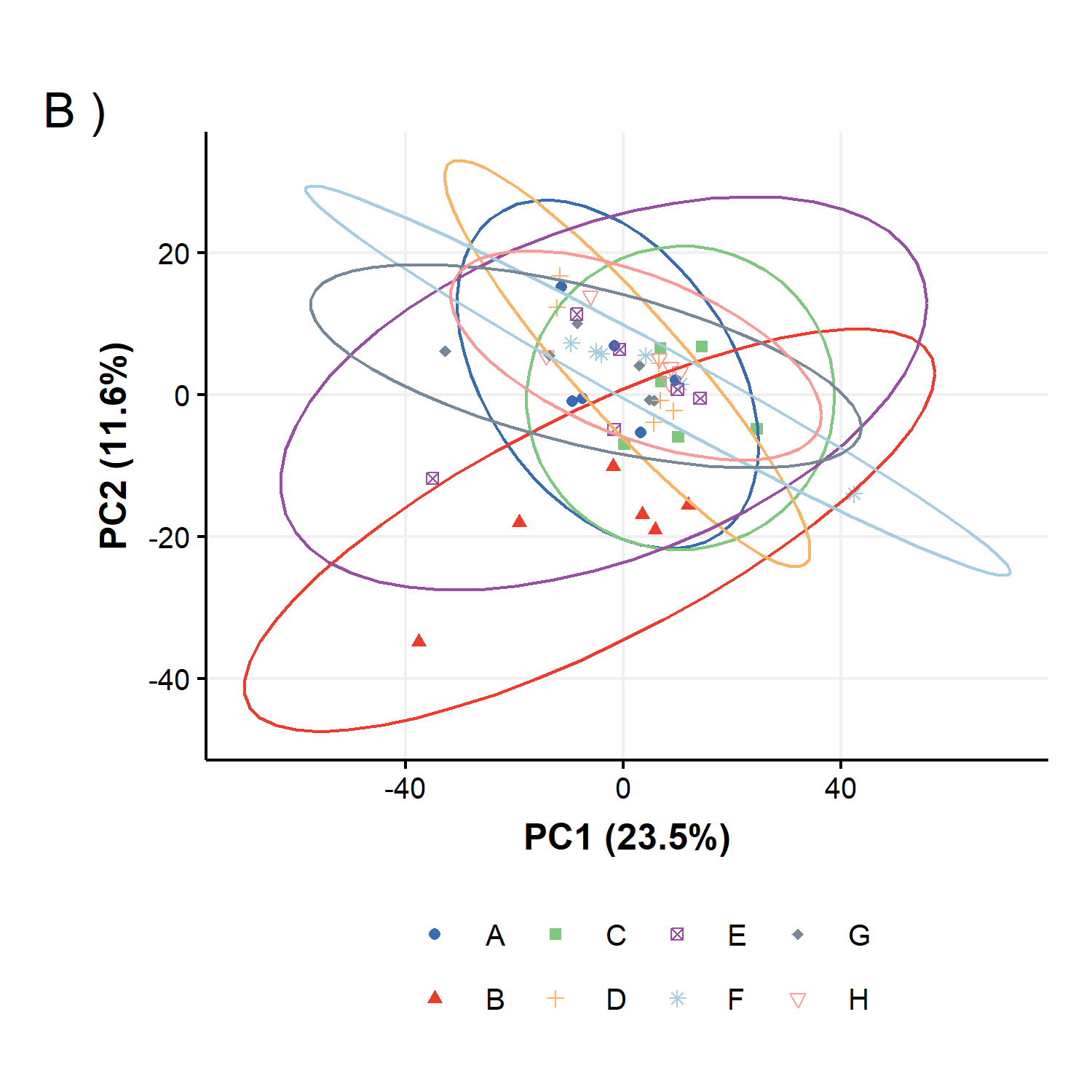
), Fe_3_O_4_ (high dose) (square
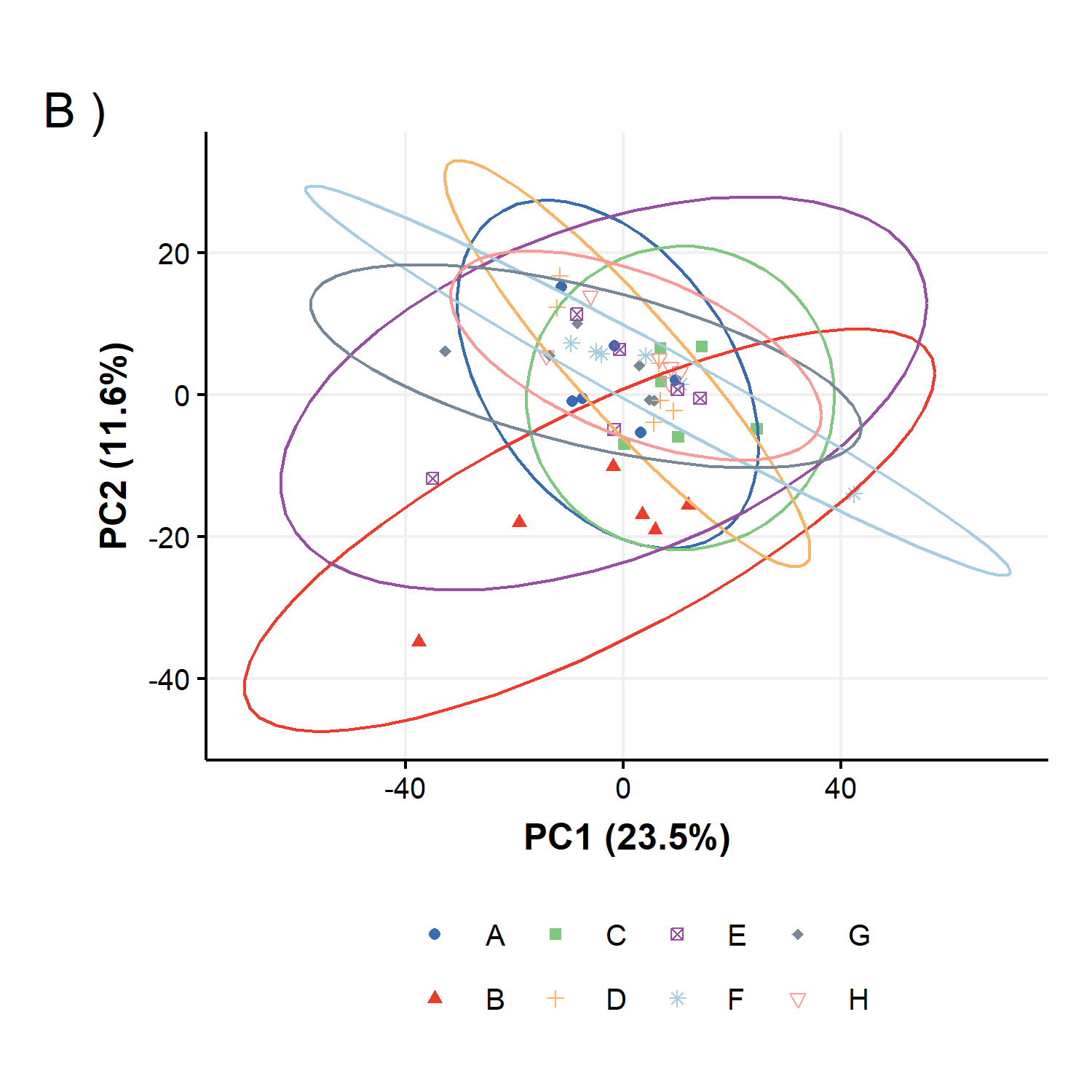
), and FeOx-mix (high dose) (plus
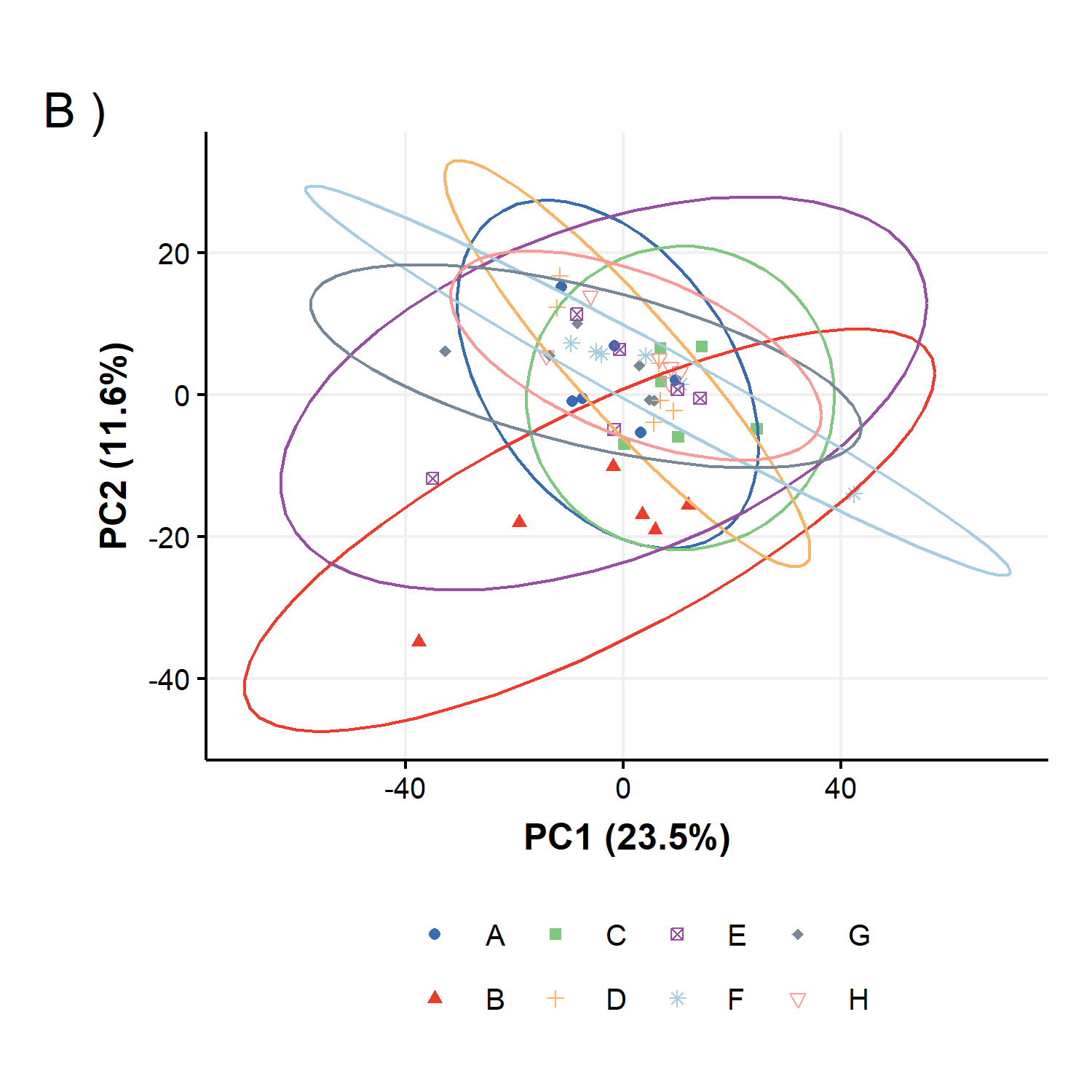
) at 1 day and 7 days post-exposure.
