# Supplementary material for: Environmentally Relevant Iron Oxide Nanoparticles Produce Limited Acute Pulmonary Effects in Rats at Realistic Exposure Levels
Source: Int J Mol Sci. 2021 Jan 8;22(2):556. doi: 10.3390/ijms22020556 (PMC7827273; doi:10.3390/ijms22020556)
Supplement: Supplementary file 1 [file ijms-22-00556-s001.zip › Supplementary Information_1 HRV study_with track changes for proofs.docx]

**Environmentally relevant iron oxide nanoparticles produce limited acute pulmonary effects in rats at realistic exposure levels**

Chang Guo, Ralf J. M. Weber, Alison Buckley, Julie Mazzolini, Sarah Robertson, Juana Maria Delgado-Saborit, Joshua Z. Rappoport, James Warren, Alan Hodgson, Paul Sanderson, James Kevin Chipman, Mark R. Viant, Rachel Smith

**SUPPLEMENTARY INFORMATION_1**

**Preliminary study on the effect of exposure to a Fe_3_O_4_NP aerosol on Heart Rate Variability**

**1. Introduction**

Studies in humans have indicated associations between measures of heart rate variability (HRV) and adverse cardiovascular effects, and links between changes to HRV and particle inhalation have been demonstrated ^1^. Following these, a number of studies have been undertaken to explore the effects of the particle, including nanoparticle, inhalation on HRV in animal models and investigate the potential mechanisms for the effects seen, e.g. Upadhyay, Stoeger (2); Zheng, McKinney (3); and Rossi, Savi (4). The majority of these studies have used invasive techniques to obtain electrocardiograms (ECGs) in unrestrained animals, requiring anaesthesia and surgical implantation of telemetry devices. It is possible that anaesthesia may depress cardiovascular function, and a recovery time of at least 2 weeks after transmitter implantation is required ^5^. According to several studies, physiological parameters at baseline are lower in animals implanted with transmitters than those obtained with traditional techniques requiring restraint ^6^. There are also difficulties in analysing and interpreting HRV data for unrestrained animals due to variable activity levels ^7^. To address these two issues a non-invasive technique was developed to obtain ECGs in conscious rats using foot pad electrodes within a nose-only inhalation exposure tube. The aim of the preliminary experiment described here was to determine if the inhalation of iron oxide NPs by aged rats provoked a change in HRV using this non-invasive system.

**2. Materials and Methods**

***Heart rate variability (HRV) analysis***


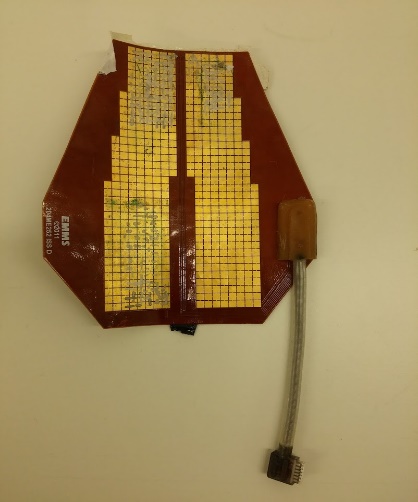
A non-invasive technique was developed to obtain Electrocardiograms (ECGs) in conscious rats when 3 paws contact 3 separate electrodes. This method is much less traumatic than implanted telemetry as it requires no surgery and avoids using anaesthesia. To obtain an ECG trace, the rat was positioned on the ECG recording platform within a nose-only inhalation system exposure tube such that each foot was located on a pad (an array) of gel-coated ECG electrodes (EMMS). Each ECG electrode comprised of a grid of wafer-thin, interlinked, gold squares photo-etched onto a pliable, non-conducting, platform (Figure 1). For each foot position, a block of appropriately positioned gold squares was isolated from the other squares by scoring and breaking the interlinking gold. Each ECG electrode was connected by shielded cables to a pre-amplifier, to boost the signal and an amplifier. The amplified signal was then digitized and recorded using specialised data acquisition software (eDacq). All electronic components and the eDacq software were supplied by EMMS (electromedsys.com).

**Figure 1.** Rat ECG foot pad electrode.

Each ECG trace was assessed using peak detection (QRS) software to identify all possible R peaks. The ECG trace, with R peaks identified, was then visually scanned to verify the quality of both the ECG signal and the accuracy of the peak detection, as time domain parameters can be affected by artefacts and outliers. The removal of artefact spikes and peaks rejected by the detection software and manually following inspection, unavoidably results in the loss of data from unknown locations in the dataset, therefore, the dataset is not a collection of contiguous RR vales, although the majority of the RR intervals are contiguous. The software package Minitab 17 (Minitab Ltd., Coventry, UK) was used with each dataset to display a time series plot of the data from each animal (Figure 2) and determine time domain HRV parameters. Kubios HRV 3.0 (University of Kuopio, Kuopio, Finland) was used to investigate frequency domain parameters.


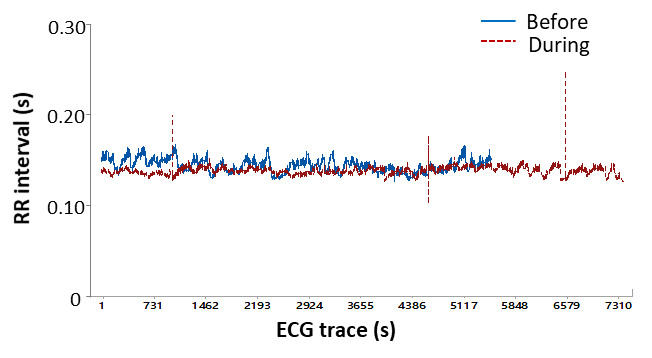

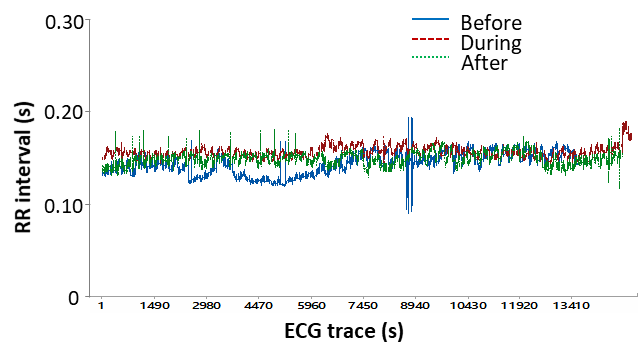


**Figure 2.** Representative time series plot of ECG trace on two separate occasions (tube, in chamber) for the control group (left) and on three separate occasions (before exposure, during exposure and after exposure) for the Fe_3_O_4_NP exposure group (right).

Two commonly reported time domain parameters, SDNN and RMSSD, were determined. These are typically expressed in milliseconds, where SDNN is the standard deviation of the (normal-to-normal) R-peak intervals and RMSSD is the root mean square of successive differences. In addition, three frequency domain parameters were determined, corresponding to spectral power in the high frequency (HF; 0.78 – 2.5 Hz), low frequency (LF; 0.19 – 0.74 Hz) and very low frequency (VLF; 0 – 0.18 Hz) ranges.

***Animals***

All rats were housed in conventional conditions, breathed unfiltered air, and were fed standard balanced food and water ad libitum, as described in the main text. All rats (female, Wistar-Kyoto, 18 months at time of exposure) were acclimatised to a nose-only inhalation restraint tube prior to exposure. Ethical approvals for experiments are outlined in main text.

***Exposure system and aerosol characterisation***

The exposure system and aerosol characterisation were as described in the main text.

***Experimental design***

Three rats were exposed for 1 hr by nose-only inhalation to a Fe_3_O_4_NP aerosol (4 mg/m^3^) generated by compressed air nebulisation of a Fe_3_O_4_NP suspension. ECG recordings were taken before, during and after the exposure such that each rat was its own control. Two rats were used as system controls and ECG recordings were collected just before and during a 1 hr exposure to clean air.

**3. Results**

Aerosol characterisation results and estimates of deposited dose are presented in Table 1.

**Table 1.** Summary of aerosol characteristics and deposited dose estimates.

| **Exposure group** | **CMD (nm)** | **GSD** | **Number conc. (particles/cm^3^)** | **Mass conc. (mg/m^3^)** | **Total lung dose (µg)** |
| --- | --- | --- | --- | --- | --- |
| Fe_3_O_4_NP HRV | 151 | 2.00 | 2.19 x 10^5^ | 4.1 | 13.3 |
| HRV air control | 14 | 1.50 | 2.27 x 10^4^ | 0 | 0 |

Heart rate (HR) and HRV results as a percentage of pre-exposure values are shown in Table 2. The average HR for the 3 rats exposed to Fe_3_O_4_NP aerosols was 427 ± 26 bpm before exposure, 387 ± 31 bpm during the exposure and 424 ± 32 bpm afterwards. The HR during the exposure was significantly lower, typically 90% of the pre- and post-exposure values, whereas the meanRR was increased. However, the average HR post-exposure was virtually identical to that determined pre-exposure suggesting that limited effects had occurred.

**Table 2.** Heart rate and HRV responses to inhaled Fe_3_O_4_NPs in aged rats (expressed as percentage of values before exposure).

| Exposed Group | Conditions |  | **Mean RR** | **SDNN** | **Heart Rate** | **RMSSD** | **VLF**  **(ms^2^)** | **LF**  **(ms^2^)** | **HF**  **(ms^2^)** |
| --- | --- | --- | --- | --- | --- | --- | --- | --- | --- |
| Air | During (n=2) | mean | 104.8% | 103.19% | 95.9% | 112.7% | 90.1% | 109.0% | 140.8% |
|  |  | s.e.m. | 6.4% | 20.18% | 6.0% | 27.2% | 38.86% | 35.0% | 30.2% |
| Fe_3_O_4_NPs | During (n=3) | mean | 109.9% | 112.9% | 90.8% | 118.6% | 109.0% | 145.5% | 122.7% |
|  |  | s.e.m. | 1.0% | 16.3% | 0.8% | 13.3% | 26.1% | 54.4% | 31.4% |
|  | After (n=3) | mean | 100.4% | 98.4% | 99.5% | 93.5% | 102.3% | 109.0% | 78.1% |
|  |  | s.e.m. | 1.4% | 4.9% | 1.5% | 2.7% | 11.7% | 9.7% | 11.8% |

The HRV parameter values showed no statistically significant differences between exposed and control and pre-, and post-exposure. Variability between animals was evident, for example, RMSDD data obtained for the same animal showed an unclear pattern when comparing the mean RMSDD values for the ‘before’ exposure with the mean RMSDD values ‘during’ or ‘after’ exposure in the individual rats (Table 3). Further, there was no significant change to SDNN during exposure to Fe_3_O_4_NP aerosols.

**Table 3.** Comparison of individual animal RMSSD results for animals exposed to Fe_3_O_4_NPs.

| Compared condition | Higher RMSDD (indicative of better HRV status) | | |
| --- | --- | --- | --- |
|  | Rat 1 | Rat 2 | Rat 3 |
| Before/After | Before | No difference | Before |
| Before/During | Before | During | During |

**Discussion and Conclusions**

This preliminary study on the effects of inhalation of Fe_3_O_4_ NPs on aged rats using a novel implant-free system found no consistent effects of exposure on HRV. The results contrast with those from other studies reported in the literature. For example, a study using an implanted system in aged spontaneously hypertensive rats found no change in comparison to control in heart rate, SDNN or RMSSD during a 24 h exposure to an ultrafine carbon aerosol (180 µg/m^3^), but significant changes in SDNN and LF/HF were found at some post-exposure times ^2, 3^ exposed rats for 5 h to a carbon nanotube aerosol (5 mg/m^3^) and, using an implanted telemetry system, found no significant difference in HR and HRV parameter values at post-exposure times between exposed and control, but significant changes in some parameters during exposure (RMSSD, LF, HF). In a study using instillation of TiO_2_ NPs in spontaneously hypertensive rats no significant changes in RMSSD were found following 6 weeks instillation of 2mg/kg per week, but significant changes in SDNN and parameters associated with arrhythmia were seen at weeks 6 and 7 ^4^. However, it is difficult to compare these study results directly because of the significant methodological differences and it is acknowledged that the small sample numbers in our preliminary study provide limited statistical power for our results.

As noted above there are difficulties in analysing data from unrestrained rats, however, some studies have shown that restraining an animal can acutely affect heart rate, blood pressure, body temperature, endogenous hormone release and body weight, and possibly lower the threshold for arrhythmia ^6, 8^. We attempted to negate and/or attenuate such effects by prior acclimatisation of the animals to the restraint tubes and allowing a period of acclimation prior to recording or analysing the data. Typically, the rats were calm within the exposure tubes following initial loading, primarily resting/sleeping.

In general, use of the system was straightforward, and it is hoped that additional studies will be possible in the future to further characterise the system and investigate the potential effects on HRV of NP inhalation using this novel system with its practical and flexible benefits in terms of the lack of a requirement for implanted telemetry with the associated reduction in effort and costs.

**4. References**

1. Rowan WH, Campen MJ, Wichers LB, Watkinson WP. Heart rate variability in rodents: uses and caveats in toxicological studies. Cardiovascular Toxicology. 2007;7(1):28-51.

2. Upadhyay S, Stoeger T, George L, Schladweiler MC, Kodavanti U, Ganguly K, Schulz H. Ultrafine carbon particle mediated cardiovascular impairment of aged spontaneously hypertensive rats. Particle and Fibre Toxicology. 2014;11(1):36.

3. Zheng W, McKinney W, Kashon M, Salmen R, Castranova V, Kan H. The influence of inhaled multi-walled carbon nanotubes on the autonomic nervous system. Particle and Fibre Toxicology. 2016;13(1):8.

4. Rossi S, Savi M, Mazzola M, Pinelli S, Alinovi R, Gennaccaro L, Pagliaro A, Meraviglia V, Galetti M, Lozano-Garcia O, Rossini A, Frati C, Falco A, Quaini F, Bocchi L, Stilli D, Lucas S, Goldoni M, Macchi E, Mutti A, Miragoli M. Subchronic exposure to titanium dioxide nanoparticles modifies cardiac structure and performance in spontaneously hypertensive rats. Particle and Fibre Toxicology. 2019;16(1):25.

5. Chu V, Otero JM, Lopez O, Morgan JP, Amende I, Hampton TG. Method for non-invasively recording electrocardiograms in conscious mice. BMC Physiol. 2001;1:6.

6. Farraj AK, Hazari MS, Haykal-Coates N, Lamb C, Winsett DW, Ge Y, Ledbetter AD, Carll AP, Bruno M, Ghio A, Costa DL. ST depression, arrhythmia, vagal dominance, and reduced cardiac micro-RNA in particulate-exposed rats. Am J Respir Cell Mol Biol. 2011;44(2):185-96.

7. Couderc JP, Elder A, Cox C, Zareba W, Oberdorster G, editors. Limitations of power-spectrum and time-domain analysis of heart rate variability in short-term ECG recorded using telemetry in unrestrained rats. Computers in Cardiology; 2002 22-25 Sept. 2002.

8. Kramer K, van Acker SA, Voss HP, Grimbergen JA, van der Vijgh WJ, Bast A. Use of telemetry to record electrocardiogram and heart rate in freely moving mice. J Pharmacol Toxicol Methods. 1993;30(4):209-15.
