# Supplementary material for: Environmentally Relevant Iron Oxide Nanoparticles Produce Limited Acute Pulmonary Effects in Rats at Realistic Exposure Levels
Source: Int J Mol Sci. 2021 Jan 8;22(2):556. doi: 10.3390/ijms22020556 (PMC7827273; doi:10.3390/ijms22020556)
Supplement: Supplementary file 1 [file ijms-22-00556-s001.zip › Supplementary Information_2 FeOx mixture ratio calculations and additional dose metrics.docx]

**Environmentally relevant iron oxide nanoparticles produce limited acute pulmonary toxicity at realistic doses**

Chang Guo, Ralf J. M. Weber, Alison Buckley, Julie Mazzolini, Sarah Robertson, Juana Maria Delgado-Saborit, Joshua Z. Rappoport, James Warren, Alan Hodgson, Paul Sanderson, James Kevin Chipman, Mark R. Viant, Rachel Smith

**SUPPLEMENTARY INFORMATION_2**

**FeOx mixture ratio calculations and additional dose metrics**

1. **FeOx mixture ratio calculations**

From their roadside investigations, Sanderson et al ^1^ estimated that the % distribution of nano-sized primary particles of the three main iron oxides observed in the ultrafine fraction of the roadside aerosol was as follows: 32% Fe_3_O_4_, 44% γ-Fe_2_O_3_ and 20% α-Fe_2_O_3_. For the in vitro investigations, a nanoparticle suspension with equivalent number-based ratios of the three main iron oxides was made, based on the calculations detailed below. To create an aerosol of agglomerates containing similar ratios of primary particles, dispersions of each type of Fe oxide where first mixed in the correct ratios and then aerosolised.

Stock solutions of mass concentrations *C_Fe3O4_*, *C_γ-Fe2O3_* and *C_α-Fe2O3_* of Fe_3_O_4_, γ-Fe_2_O_3_ and α-Fe_2_O_3_, respectively, were provided by Promethean Particles Ltd. The number concentration of primary particles, *N_pp_*, in a given stock solution is related to the mass concentration, *C*, as follows:

$$N_{pp}=\frac{C}{m_{pp}} (1)$$

where *m_pp_* is the mass of a single nanoparticle. Assuming all primary particles are spheres with average radius, *r_pp_*, and bulk density, *ρ*, then the mass of a single nanoparticle is:

$$m_{pp}=\rho v_{pp} (2)$$

where the volume of a single spherical primary particle, *v_pp_*, is calculated as

$$v_{pp}=\frac{4}{3}\pi{r_{pp}}^{3} (3)$$

Substituting equations (2) and (3) into equation (1), the number concentration of a given nanoparticle suspension can be calculated using:

$$N_{pp}=\frac{C}{\rho\frac{4}{3}\pi{r_{pp}}^{3}} (4)$$

The concentration of primary particles, *N_pp_*, can also be written as:

$$N_{pp}=\frac{N}{V} (5)$$

Where *N* is the total number of primary particles in solution and *V* is the volume of the solution. Combining equations (4) and (5):

$$V=\frac{N\rho\frac{4}{3}\pi{r_{pp}}^{3}}{C} (6)$$

Knowing the mass concentration, *C,* of each iron oxide nanoparticle stock solution, the ratios of volumes of each stock solution required to give the desired number-based ratios of 1.6:2.2:1.0 for Fe_3_O_4_: γ-Fe_2_O_3_: α-Fe_2_O_3_ can be calculated using the following

$$\frac{V_{\alpha-{Fe}_{2}O_{3}}}{V_{\gamma-{Fe}_{2}O_{3}}}=\frac{N_{\alpha-{Fe}_{2}O_{3}}}{N_{\gamma-{Fe}_{2}O_{3}}}.\frac{\rho_{\alpha-{Fe}_{2}O_{3}}}{\rho_{\gamma-{Fe}_{2}O_{3}}}.\frac{{r_{\alpha-{Fe}_{2}O_{3}}}^{3}}{{r_{\gamma-{Fe}_{2}O_{3}}}^{3}}.\frac{C_{\gamma-{Fe}_{2}O_{3}}}{C_{\alpha-{Fe}_{2}O_{3}}} (7)$$

$$\frac{V_{{Fe}_{3}O_{4}}}{V_{\gamma-{Fe}_{2}O_{3}}}=\frac{N_{{Fe}_{3}O_{4}}}{N_{\gamma-{Fe}_{2}O_{3}}}.\frac{\rho_{{Fe}_{3}O_{4}}}{\rho_{\gamma-{Fe}_{2}O_{3}}}.\frac{{r_{{Fe}_{3}O_{4}}}^{3}}{{r_{\gamma-{Fe}_{2}O_{3}}}^{3}}.\frac{C_{\gamma-{Fe}_{2}O_{3}}}{C_{{Fe}_{3}O_{4}}} (8)$$

Using data on desired number ratios, bulk densities, radii, and suspension concentrations (Table SA), the equations above can be simplified as follows for the in vitro experiments:

$$V_{\alpha-{Fe}_{2}O_{3}}=3.54 V_{\gamma-{Fe}_{2}O_{3}} (9)$$

$$V_{{Fe}_{3}O_{4}}=33.69 V_{\gamma-{Fe}_{2}O_{3}} (10)$$

Similarly, for the in vivo investigation:

$$V_{\alpha-{Fe}_{2}O_{3}}=3.54 V_{\gamma-{Fe}_{2}O_{3}} (11)$$

$$V_{{Fe}_{3}O_{4}}=18.72 V_{\gamma-{Fe}_{2}O_{3}} (12)$$

Thus volumes were combined in the following ratios for the in vitro experiments,

$$V_{\gamma-{Fe}_{2}O_{3}}: V_{\alpha-{Fe}_{2}O_{3}}:V_{{Fe}_{3}O_{4}}=1 :3.54 :33.69 (13)$$

and for the in vivo experiments:

$$V_{\gamma-{Fe}_{2}O_{3}}: V_{\alpha-{Fe}_{2}O_{3}}:V_{{Fe}_{3}O_{4}}=1 :3.54 :18.72 (14)$$

**Table SA** Iron Oxide suspension properties.

| Material | Primary particle radius^†^ (nm) | Bulk density^‡^ (gcm^-3^) | Ratio of NPs in environment by number^§^ (normalized to α-Fe_2_O_3_) | C in vitro (mg/ml) | C in vivo (mg/ml) |
| --- | --- | --- | --- | --- | --- |
| Fe_3_O_4_ | 9.71 | 5.1 | 1.6 | 10 | 18 |

| γ-Fe_2_O_3_ | 2.74 | 4.9 | 2.2 | 10 | 10 |
| --- | --- | --- | --- | --- | --- |

| α-Fe_2_O_3_ | 5.31 | 5.24 | 1.0 | 10 | 10 |
| --- | --- | --- | --- | --- | --- |

^†^see main text, ^‡^manufacturers data, ^§^Saunders et al ^1^

1. **Additional Dose Metrics**

While mass has traditionally been used as the biologically effective dose metric for soluble toxins, for non- or poorly-soluble nanoparticles, a category which a lot of nanoparticles fall into, where only the surface of the particle can interact with biological fluids and tissues, surface area, particle number and volume have all been suggested as more relevant dose metrics ^2-7^. Schmid and Stoeger^8^ provides a useful summary on the matter. While there is no consensus on the most relevant dose metric to use, and with the answer being unlikely to be a simple, single, “catch-all” metric, where possible mass, surface area, number and volume should be reported. Details are given below of the particle number, surface area and volume calculations used in this study.

***Particle Number***

Agglomerate number concentrations are measured directly, primary particle number concentrations are more complex to determine. For the aerosols delivered in the inhalation study, two methods have been used to calculate the primary particle number concentration: one based on the measured mass concentration^8^ and the other using the mobility equivalent size distribution measured by SMPS suggested by Lall and Friedlander ^9^. Both require accurate knowledge of the primary particle diameter from TEM measurements.

The first method uses the average primary particle diameter, *d_p_*, measured from TEM images and the average mass concentration, *M*, calculated from gravimetric measurements, and assuming all primary particles are spherical and have inherent material density, *ρ*, the primary particle concentration, *N_p_*, can be estimated as follows:

$$N_{p}=\frac{6M}{\pi\rho d_{p}^{3}}$$

For comparison, *N_p_*, was also estimated from the aerosol size distribution and primary particle diameter. Lall and Friedlander ^9^ related the number of primary particles per agglomerate, *N*, to mobility size for agglomerates, *d_m_*, using the following expression:

$$N=\frac{d_{m}}{C(d_{m})}\frac{12\pi\lambda}{c^{*}d_{p}^{2}}$$

Where *λ* is the mean free path of gas, *C(d_m_)* the Cunningham slip correction factor and *c** the dimensionless drag force, which for aggregates oriented parallel to their relative motion is equal to 6.62. Applying this to each size bin of the measured mobility equivalent size distribution and summing over the distribution, the primary particle concentration can be estimated.

***Surface Area***

Surface area (SA) is of particular interest for ultrafine/nanoparticles due to their large surface area to mass ratio with numerous studies supporting surface area as an effective dose metric ^2, 8, 10-15^. Surface area is a difficult quantity to define and measure though, with various definitions and methods used in the literature.

Historically, the most commonly used method to measure the particulate surface area available for interaction in toxicological studies has been BET^16^, which measures mass- specific surface area based on the physical adsorption of gas molecules, most commonly Nitrogen. This method is only appropriate for relatively large quantities of powder though, which in toxicology studies may not be available and in the case of inhalation exposures may not be representative of the material when in aerosol form. As the iron oxide particles used in this study were provided in aqueous suspension, BET was not possible as a method of surface area calculation. For comparison to previous works reported in the literature though, mass-specific surface areas shall be calculated.

Other instruments developed more recently use diffusion charging with subsequent measurement of particle induced electric current to measure particle surface area^17, 18^. These measure “active (or Fuchs) surface area”, which refers to the part of the surface that causes friction in the carrier gas^19^. Particles with the same mobility diameter, whether a chain agglomerate or sphere, have the same active surface area. While active surface area is not generally considered to be biologically relevant, this type of instrument has shown promise for measuring non-porous spherical particles <100nm where active surface area is equivalent to geometric surface area. As size increases though, the relationship between active SA and geometric SA diverge.

Active surface area can also be estimated from a SMPS size distribution for compact non-spherical particles^20^. In this study, the SMPS software was used to obtain the surface area concentration from the measured number size distribution. The surface area concentration, calculation assumes that all the particles are perfect spheres and is calculated by:

$$dS=dN\cdot\pi{D_{p}}^{2}$$

Where *D_p_* is the geometric midpoint of the particle size channel. Total surface area concentration can be obtained by summing all the SMPS size channels.

Geometric surface area describes the total surface area of the primary particles. For spherical non-porous particles, it is similar to BET surface area. Schmid and Stoeger^8^ found no significant difference in the BET or geometric surface area dose-response relationships for a given material. As particle shape and structure deviates from that of smooth, compact particles, BET and geometric surface area diverges. Using estimated values of *N_p_*, the volume concentration, *V*, can be calculated as,

$$V=N_{p}\frac{\pi{d_{p}}^{3}}{6}$$

and the geometric surface area concentration, *SA_g_*, as,

$${SA}_{g}=\pi{d_{p}}^{2}N_{p}$$

From this, the volume specific surface area, *SA_V_* (m^2^/cm^3^) and mass specific surface area, *SA_M_* (m^2^/g) can be calculated using the following equations respectively.

$${SA}_{V}=\frac{SA}{V}$$

$${SA}_{M}=\frac{SA}{M}$$

For the iron oxide mixture aerosol, the number of primary particles, volume concentration and surface area concentration were calculated for each type of iron oxide separately assuming the known number-weighted mixture ratios and then summed.

1. **References**
2. Sanderson P, Su SS, Chang ITH, Delgado Saborit JM, Kepaptsoglou DM, Weber RJM, Harrison RM. Characterisation of iron-rich atmospheric submicrometre particles in the roadside environment. *Atmos Env*, 2016; 140: 167-175.
3. Oberdorster G. Significance of particle parameters in the evaluation of exposure-dose-response relationships of inhaled particles. *Inhal Toxicol*, 1996; 8 (Suppl): 73-89.
4. Maynard AD, Kuempel ED. Airborne nanostructured particles and occupational health. *J Nanopart Res.* 2005; *7*(6): 587-614.
5. Wittmaack K. In search of the most relevant parameter for quantifying lung inflammatory response to nanoparticle exposure: particle number, surface area, or what? *Environ Health Perspect*. 2007; 115(2); 187-194.
6. Pauluhn J. Poorly soluble particulates: Searching for a unifying denominator of nanoparticles and fine particles for DNEL estimation. *Toxicology*. 2011; 279 (1–3); 176-188.
7. Gebel T. Small difference in carcinogenic potency between GBP nanomaterials and GBP micromaterials. *Arch Toxicol.* 2012; *86*(7): 995-1007.
8. Donaldson K, Schinwald A, Murphy F, Cho WS, Duffin R, Tran L et al, Poland C. **The biologically effective dose in inhalation nanotoxicology.** *Acc Chem Res.* 2013; 46(3): 723-732.
9. Schmid O, Stoeger T. Surface area is the biologically most effective dose metric for acute nanoparticle toxicity in the lung. *J Aerosol Sci.* 2016; 99: 133-143.
10. Lall AA, Friedlander SK. On-Line Measurement of Ultrafine Aggregate Surface Area and Volume Distributions by Electrical Mobility Analysis: 1. Theoretical Analysis. *J Aerosol Sci.* 2006; 37: 260-271.
11. Hadrup N, Saber AT, Kyjovska ZO, Jacobsen NR, Vippola M, Sarlin E, Ding Y, Schmid O, Wallin H, Jensen KA, Vogel U. Pulmonary toxicity of Fe_2_O_3_, ZnFe_2_O_4_, NiFe_2_O_4_ and NiZnFe_4_O_8_ nanomaterials: Inflammation and DNA strand breaks. *Environ Toxicol Pharmacol.* 2020; 74: 103303.
12. Roach KA, Anderson SE, Stefaniak AB, Shane HL, Kodali V, Kashon M, Roberts JR. Surface area-and mass-based comparison of fine and ultrafine nickel oxide lung toxicity and augmentation of allergic response in an ovalbumin asthma model. *Inhal Toxicol.* 2019; 31(8): 299-324.
13. Tran CL, Buchanan D, Cullen RT, Searl A, Jones AD, Donaldson K. Inhalation of poorly soluble particles. II. Influence of particle surface area on inflammation and clearance. *Inhal Toxicol.* 2000; 12(12): 1113-1126.
14. Danielsen PH, Knudsen KB, Štrancar J, Umek P, Koklič T, Garvas M, Vanhala E, Savukoski S, Ding Y, Madsen AM, Jacobsen NR. Effects of physicochemical properties of TiO2 nanomaterials for pulmonary inflammation, acute phase response and alveolar proteinosis in intratracheally exposed mice. *Toxicol Appl Pharmacol*. 2020; 386: 114830.
15. Waters KM, Masiello LM, Zangar RC, Tarasevich BJ, Karin NJ, Quesenberry RD, Bandyopadhyay S, Teeguarden JG, Pounds JG, Thrall BD. Macrophage Responses to Silica Nanoparticles are Highly Conserved Across Particle Sizes. *Toxicol Sci.* 2009; 107(2): 553–569.
16. Moshammer H, Neuberger M. The active surface of suspended particles as a predictor of lung function and pulmonary symptoms in Austrian school children. *Atmos Environ.* 2003; *37*(13): 1737-1744.
17. Brunauer S, Emmett PH, Teller E. Adsorption of gases in multimolecular layers. *J Am Chem Soc.* 1938; 60(2): 309-319.
18. Fissan H, Neumann S, Trampe A, Pui DYH, Shin WG. Rationale and principle of an instrument measuring lung deposited nanoparticle surface area. *J Nanoparticle Res*. 2007; 9: 53-59.
19. Shin WG, Pui DYH, Fissan H, Neumann S, Trampe A. Calibration and numerical simulation of nanoparticle surface area monitor (TSI model 3550 NSAM). *J Nanoparticle Res*. 2007; 9: 61-69.
20. Keller A, Fierz M, Siegmann K, Siegmann HC, Filippov A. Surface science with nanosized particles in a carrier gas. *J Vac Sci Tech A*. 2001; 19(1): 1-8.
21. Ku BK, Evans DE. Investigation of aerosol surface area estimation from number and mass concentration measurements: Particle density effect. *Aerosol Sci Tech.* 2012; 46(4): 473-484.
