# Supplementary material for: Environmentally Relevant Iron Oxide Nanoparticles Produce Limited Acute Pulmonary Effects in Rats at Realistic Exposure Levels
Source: Int J Mol Sci. 2021 Jan 8;22(2):556. doi: 10.3390/ijms22020556 (PMC7827273; doi:10.3390/ijms22020556)
Supplement: Supplementary file 1 [file ijms-22-00556-s001.zip › Supplementary Tables.docx]

**Environmentally relevant iron oxide nanoparticles produce limited acute pulmonary effects in rats at realistic exposure levels**

Chang Guo, Ralf J. M. Weber, Alison Buckley, Julie Mazzolini, Sarah Robertson, Juana Maria Delgado-Saborit, Joshua Z. Rappoport, James Warren, Alan Hodgson, Paul Sanderson, James Kevin Chipman, Mark R. Viant, Rachel Smith

**SUPPLEMENTARY Tables**

**Table S1** FeOxNP inhalation studies.

| **Origin FeOx particles** | **Characterization of primary particles** | **Characterization of aerosols**  (CMD - count median diameter; MMAD - mass median aerodynamic diameter; GMD - geometric mean diameter; GSD - geometric standard deviation) | **Exposure dose**  **(aerosol concentration × exposure duration)** | **Animal models** | **Animal sacrifice time points post-exposure** | **Brief biological findings** | **Reference**  (^†^ nose-only inhalation;  ^††^ whole-body exposure) |
| --- | --- | --- | --- | --- | --- | --- | --- |
| Dust with FeOx w/ SiO_2_ | - | - | 4 h/d × 5d/w × 52 wks | Mice (strain unknown) | Span of life | The iron oxide dust also produced a definite increase in the incidence and malignancy of the lung tumors in mice. | ^1^ |
| Dust with FeOx (81.5) | - | < 1 µm (88%) | 2.3-7.8 mg/m^3^ (Lewinski *et al.*, 2013) × 3 h/d × 140 d | Rats | 80, 235 d post | The radiographs showed pin-point dense shadows located at the periphery of the lung field and corresponding with small collections of iron oxide either in the pleura or just beneath the surface of the lung. No intensification of the lung markings was apparent. | ^2^ |
| ^59^FeOx | - | 0.09 µm (CMD, GSD 1.8) | 30 mg/m^3^ × 60 min | Beagle dogs | Up to 6 months post | The biological half-time for alveolar clearance ranged from 44 to 82 days with an average value of 62 days (sd 8.8 days), which suggests the probability of a normal clearance rate and the possibility of its use as a functional test. | ^3†^ |
| ^59^FeO_x_ | - | 0.068 µm (CMD, GSD 1.62) | 300-900 mg/m^3^ × 45 min | Rochester rats | Up to 30 d post | Upper and lower respiratory tract retention has been presented in two ways. The differences in clearance rates thus obtained are due to a significant early absorption or transport as reflected in carcass values. | ^4†^ |
| FeOx | - | 0.3 µm (MMAD, GSD 1.8) | 700 mg/m^3^ × 16, 30, 235 min | Albino rats | Up to 10 d post | The deposition factors found for the examined FeOx particles om rats were similar to those proposed by ICRP for man. The phase II component of the pulmonary clearance was of shorter half time than that in man. | ^5^ |
| FeOx | - | 0.1 µm (d), 0.1-3 (SEM) | 170-200 mg/m^3^ × 3 h | CD-1 mice | Up to 14 months post | The iron particles settle extensively but not uniformly on pulmonary alveolar surfaces. Clearance is centripetal and involves an extracellular mechanism fed by fluid currents sweeping across the surface, and a cellular mechanism principally involving alveolar macrophages. | ^6^ |
| FeOx | 5 nm | 0.15 nm (MMAD), GSD = 2.2 | 300 mg/m^3^ × 3 h | CD-1 mice | 1, 4, and 7 days post | Iron oxide was pinocytosed and converted to ferritin and hemosiderin in all epithelial cell types except mucous cells. Iron content increased over time and approximately 50% of the nonmucous cells contained hemosiderin by 4 days postexposure. Ferritin and hemosiderin, but not iron oxide, were noted in connective tissue cells in the submucosa beneath the airway epithelium. Soluble iron and/or ferritin produced in the airway epithelial layer was transported to the submucosa, but normal epithelium prevented the penetration of deposited iron oxide particles to the connective tissue compartment. | ^7^ |
| ^59^Fe_3_O_4_ (as inert tracer particles) | - | 1.5 (GSD) | 15.4±4.5 mg/m^3^ × 2 h | Fisher 344 rats | Up to 120 d post | ^59^Fe_3_O_4_ retention on day 120 was significantly lower in all exposure groups (coal mine dust, diesel exhaust, coal mine due plus diesel exhaust) as compared to controls. Short term clearance determined from Fe_3_O_4_ retention of day 1 was not significantly different between the groups. | ^8†^ |
| γ-Fe_2_O_3_ | - | 0.155 µm (TEM), 0.73 µm (MMAD, GSD 1.26) | 200-300 mg/m^3^ × 0.5-1 h | New Zealand white rabbits | 1, 10, 40 d post | The rate of relaxation changed with time after particle inhalation, especially during the first day; changes in the relaxation rate correlated with an estimate of in situ particle phagocytosis during that time. | ^9†^ |
| γ-Fe_2_O_3_ | - | 0.072 µm (0.02-0.14, TEM) | 57 and 90 μg/m^3^ × 6 h/d × 3 d | Sprague-Dawley (SD) rats | Post | Inhalation of iron particles leads to oxidative stress associated with a proinflammatory response in a dose‐dependent manner. The activation of NFκB may be involved in iron‐induced respiratory responses. | ^10††^ |
| γ- Fe_2_O_3_ | 25±2 nm (TEM)  BET 17±1 m^2^/g | 0.2 µm (SMPS, GSD 1.3) | 7.62 mg/m^3^ x 4 h, 3.552 mg/m^3^ × 4 h/d × 5d/w × 2 wks | C57Bl/6 mice | 0, 3 wks post | No significant pathology was found following acute exposure. Immediately following sub-acute exposure, iron NP-exposed mice showed increased inflammation compared to sentinels, same as copper NP-exposed. Three weeks post-exposure, all inflammatory markers decreased for iron NP-exposed mice, but some remained elevated for copper NP-exposed mice. | ^11††^ |
| Fe_3_O_4_ (FeREX) | 10 nm (manufact.) | 5.6 ± 0.8 *μ*m (MMAD, GSD 1.30 ± 0.03) | 140 mg/m^3^ × 2 h | BALl/6 mice | 0 d Post | Regional concentrations of iron in the lungs of the mice ranged from 1.1 ± 0.8 *μ*g/cm^3^ in peripheral lung regions to 2.7 ± 1.4 *μ*g/cm^3^ in the central lung, with no significant difference between the left and right lung. MRI shows promise for *in vivo* measurement of regional lung concentrations of superparamagnetic iron oxide nanoparticles | ^12†^ |
| Fluorescent FeOx | 9 nm (TEM) | 49 nm (GMD, GSD 1.8) (low conc.); 51 nm (GMD, GSD 1.7) (high conc.) | 0.16-0.32 mg/m^3^ × 4 h/d × 5d/w × 4 wks | Slc:ICP mice | 0 d Post | Fluorescent FeOx NPs were distributed (by MRI and CLSM) in various organs, including the liver, testis, spleen, lung and brain. T2-weighted spin-echo MR images showed that FMNPs could penetrate the blood-brain-barrier (BBB). | ^13†^ |
|  |  |  |  |  |  | The Fluorescent FeOx NPs induced extramedullary hematopoiesis in the spleen without having a pulmonary effect. | ^14^ |
| Fe_3_O_4_ (Ferroxide^®^ Black 88P) | 300–600 nm; 10.5 m^2^/g (BET) | 1.5 µm (MMAD, GSD 2.1) | 10.1 ± 1.44, 19.7 ± 3.27, 45.61 ± 6.77, and 95.84 ± 17.6 mg/m^3^ × 6 h/d × 5d/w × 4 wks | Wistar rats strain Bor:WISW (SPF-Cpb) | 10, 60, 170 d post | For the tested poorly soluble dusts, pulmonary toxicity (inflammation) corresponds best with the mass-based cumulative lung exposure dose. | ^15†^ |
| Soot or iron-soot particles (PM2.5) | 40–50 nm | 91 nm (median) and 120 nm (mean) (iron-soot particles); 210 nm (median) and 360 nm (mean) (soot particles) | 218 ± 9 μg/m^3^ (soot-only) or 211 ± 4 μg/m^3^ (iron-soot 17% ± 1% iron) X 6 h/d for 3 d | C57BL/6 mice | 1, 2 d post | Soot-only exposure decreased short-term HRV (root mean square of successive difference). With the addition of iron, all HRV parameters were significantly reduced. In nonexposed mice, vagal blockade significantly reduced all HRV parameters, suggesting that HRV is, in part, under vagal regulation in mice. Iron-soot exposure had no significant effect on resting membrane potential but decreased spiking responses of the identified cardiac vagal neurons to depolarizations (*p* < 0.05). The decreased spiking response was accompanied with a higher minimal depolarizing current required to evoke spikes and a lower peak discharge frequency. | ^16^ |
| FeOx in combination with soot | - | mixed Fe and soot 72nm (45-110 nm) | 30 and 100 µg/m^3^ (in combination with soot particles 250 µg/m^3^) X 6 h/d for 3 d | SD rats | 2 h post | Oxidative stress was observed in the form of significant elevations in GSSG and GSSG/glutathione (GSH) ratio and a reduction in ferric/reducing antioxidant power in BAL. A significant decrease in cell viability associated with significant increases in lactate LDH activity, IL-1β, and ferritin expression was noted following exposure to particles containing the highest Fe concentration. Iron from these particles was shown to be bioavailable in an in vitro assay using the physiologically relevant chelator, citrate. | ^17††^ |
| Fe_3_O_4_ | - | 22±2, 100±13, 198±31 nm (VMD); 0.61±1.8-1.07±2.03(MMAD±GSD) | 384±30 mg/m^3^ X 5 min | CD-1 mice | 0 d Post | Deposition was enhanced in the mouse lung but not in the trachea, which was consistent with the analysis of the aerodynamic time allowed for deposition and required magnetic deposition time. | ^18^ |
| Fe_3_O_4_ (Ferroxide^®^ Black 88P) | 250nm | 1.4 µm (MMAD, GSD 2) | 30, 100 mg/m^3^ (22 or 66 mg Fe/m³) × 6 h/d × 5 d/wk × 4 wks | Wistar rats | Up to 3 months post | The repeated inhalation exposure of rats to highly respirable pigment-type iron oxide caused nonspecific pulmonary inflammation which shows a clear dependence on the particle volume-dependent lung overload rather than any increased dissolution and/or bioavailability of redox-active iron. | ^19†^ |
| Fe_3_O_4_ (Ferroxide^®^ Black 88P) | - | 1.3-1.5 µm (MMAD, GSD 1.9-2.2) | 0, 4.7, 16.6, 52.1 mg/m^3^ × 6 h/d × 5 d/wk × 13 wks | Wistar rats | Up to 6 months post | The retention kinetics iron oxide reflected that of poorly soluble particles. The empirical no-observed-adverse-effect level (NOAEL) and the lower bound 95% confidence limit on the benchmark concentration (BMCL) obtained by benchmark analysis was 4.7 and 4.4 mg/m^3^, respectively, and supports an OEL (time-adjusted chronic occupational exposure level) of 2 mg/m^3^ (alveolar fraction). | ^20††^ |
| Fe_3_O_4_ (Superparamagnetic iron oxide (SPIO)) | 15-20nm (manufact.); 48 ±7 nm (SEM) | 652.1nm (DLS), 2.25 µm (GMD, GSD 2.56) | 640 mg/m^3^ × 4 h | Wistar rat | 1, 2, 14 d post | Immediately following acute exposure, rats showed increased inflammation with significantly higher levels of lavage and blood proinflammatory cytokines and were consistent throughout the observation period. Fe_3_O_4_ NPs exposure markedly increased malondialdehyde concentration, while intracellular reduced glutathione and antioxidant enzyme activities were significantly decreased in lung tissue within 24-h postexposure period. On histological observation, the lung showed an early activation of pulmonary clearance and a size-dependent biphasic nature of the Fe_3_O_4_ NPs in causing the structural alteration. | ^21†^ |
| Fe2O3 | 4-25 nm | 50 nm (FMPS, GSD 1.6) | 40-60 µg/m^3^ × 5 h | SD rats | 0 d Post | The in-vivo chemiluminescence (IVCL) measurements in the lungs of the Fe_2_O_3_ exposed animals were about 60 times higher than for the unexposed animals, indicating that the Fe_2_O_3_ test aerosol increased ROS in the lungs, as well as oxidative stress present in the heart of the animals. | ^22††^ |
| Superparamagnetic iron oxide (SPIO) (EGFP-targeted and non-targeted | - | 1.1 ± 0.1 µm (MMAD, GSD 1.9 ± 0.1) | - × 30 min | Fox Chase SCID^®^ Beige mice in orthotopic lung tumor model | 1 h, 1 wk post | Inhalation resulted in better intra-tumoral distribution compared to instillation, where most of the instilled dose resided near the major airways, with almost no particles reaching the periphery. EGFR targeting enhances tumor retention of SPIO nanoparticles while minimizing systemic exposure. Magnetic hyperthermia using targeted SPIO nanoparticles resulted in a significant inhibition of *in vivo* tumor growth. | ^23†^ |
| Fe_3_O_4_ | 12.8 nm (SEM), 88.7 m^2/^g (calculated) | 68.6 nm (CMD) | 19.9 mg/m^3^ × 4 h | Balb/c mice | 0, 6, 24, 48, 96, 168 h post | Target tissue doses of 0.009-0.4 μg/cm^2^ in lung led to an inflammatory response in the alveolar region characterized by interstitial inflammation and macrophage infiltration. Estimated in vivo macrophage SPIOnanoparticle doses ranged from 1-100 pg/cell, and induction of inflammatory markers was observed in vitro in macrophages at doses of 8-35 pg/cell. | ^24†^ |
| Fine Pigment Red 101 (Fe2O3, hematite) | 15-300 nm, 4-21 nm (TEM) | 1.1/0.6 µm (MMAD, GSD 2.5/3.2), 0.362 µm (CMD) | 29.86 ± 2.77 mg/m3 X 6 h/d X 5d | Wistar (strain Crl:WI (Han)) rats | 0 and 21 d (histopathological examinations. 3 and 24 d post (BAL toxicity) | Pigment deposition and pigment phagocytosis were observed after exposure to Pigment Red 101. Both pigments were tolerated well and caused only marginal effects in BALF or no effects at all. Only minor effects were seen on the lung by microscopic examination. There was no evidence of systemic inflammation based on acute-phase protein levels in blood. | ^25†^ |
| Coarse pigment Red 101 (predominantly Fe2O3, hematite; possibly also Fe3O4 magnetite) | 48-90 nm | 0.7/0.7 µm (MMAD, GSD 2.9/2.5), 0.255 µm (CMD) | 31.34 ± 4.49 mg/m3 X 6 h/d X 5d | Wistar (strain Crl:WI (Han)) rats  Outbred white rats | 0 and 21 d (histopathological examinations. 3 and 24 d post (BAL toxicity)  0 d Post | Pigment deposition and pigment phagocytosis were observed after exposure to Pigment Red 101. Both pigments were tolerated well and caused only marginal effects in BALF or no effects at all. Only minor effects were seen on the lung by microscopic examination. There was no evidence of systemic inflammation based on acute-phase protein levels in blood.  TEM images revealed NPs accumulated within alveolocytes and myelin sheaths of brain neural fibres. Both experimental data and mmathematical modelling showed that retention of the NPs in lungs is controlled by physiological and solubilization mechanisms | ^26†^ |
| Fe_2_O_3_ | - | 14 ± 4 nm (SEM) | ~ 1 × 4 h/d × 5 d/wk × 3, 6 or 10 m |  |  |  |  |
| iron-soot combustion particles | - | 50.4 ± 4 nm (CMD) | 38 μg/m^3^ of FeOx within 203±13 μg/m^3^ of total × 6 h/d, 5 d/wk for 5 wks | C57B6 mice | 24 h post | Inhaled ultrafine iron-soot reached the brain via the olfactory nerves and was associated with indicators of neural inflammation. | ^27††^ |

**Table S2** MPPD model input parameter values for deposition efficiency determination.

| **Parameter (unit)** | **Value** |
| --- | --- |
| Aerosol CMD (nm)^†^ | 138 |
| Aerosol GSD^†^ | 1.6 |
| Aerosol mass concentration (mg/m^3^) ^††^ | 0.05-0.5 |
| Particle density (g/cm^3^)^#^ | 2.5 |
| Aspect Ratio | 1 |
| Species | SD |
| Model/body weight (g) | Asymmetric Sprague Dawley (300 g) |
| FRC (ml) | 3.48672^##^ |
| URT volume (ml) | 0.40192^##^ |
| F (breathes/min) | 166^##^ |
| Tidal volume (ml) | 2.12614^##^ |
| Inspiration fraction | 0.5^##^ |
| Pause fraction | 0^##^ |
| Body Orientation | Stomach |
| Breathing Scenario | Nose-only Exposure |

^†^ Aerosol properties used are the averages of all iron-oxide inhalation exposures (Table 2, main text).

^††^ Aerosol mass concentrations between 0.05 and 0.5 mg/m^3^ were used in the model and had no effect on the resulting deposition efficiencies.

^#^ Particle density is the average effective density estimated from the measured size distributions and mass concentrations.

^##^ MPPD default values.

**Table S3** Additional aerosol characteristics. ^†^

| **Aerosol Parameter** | **Fe_3_O_4_ Low Dose** | **Fe_3_O_4_ High Dose** | **FeOx mix** |
| --- | --- | --- | --- |
| Primary Particle Diameter (nm) | 19.4 ± 12.5 (n=140) | 19.4 ± 12.5 (n=140) | 18.3 ± 9.6 (n=140) |
| Primary particle conc. (#/cm^3^) *derived from M, dpp & ρ^††^* | 2.45 x 10^6^ | 2.49 x 10^7^ | 7.26 x 10^7^ |
| Primary particle conc. (#/cm^3^) *Lall & Friedlander (2006)* | 4.67 x 10^5^ | 5.42 x 10^6^ | - |
| Geometric Surface Area Conc. (nm^2^/cm^3^) *derived from M, dpp &ρ* | 2.91 x 10^9^ | 2.95 x 10^10^ | 3.54 x 10^10^ |
| Active Surface Area Conc. (nm^2^/cm^3^) *Derived from SMPS measurement* | 5.23 ± 0.73 x 10^8^ | 6.46 ± 0.62 x 10^9^ | 6.47 ±1.40 x 10^9^ |
| Active surface area Conc. (nm^2^/cm^3^) *Lall & Friedlander (2006)* | 5.53 x 10^8^ | 6.43 x 10^9^ | - |
| Volume Conc. (nm^3^/cm^3^) *derived from M, dpp &ρ* | 9.41 x 10^9^ | 9.55 x 10^10^ | 9.95 x 10^9^ |
| Volume Conc. (nm^3^/cm^3^) *Lall & Friedlander (2006)* | 1.79 x 10^9^ | 2.08 x 10^10^ | - |
| Mass specific GSA (m^2^/g) *derived from M, dpp & ρ* | 61 | 61 | 72 |
| Mass specific ASA (m^2^/g) *Derived from SMPS measurement* | 12 | 13 | - |
| Volume specific GSA (m^2^/cm^3^) *derived from M, dpp & ρ* | 309 | 309 | 356 |
| Volume specific ASA (m^2^/cm^3^) *Derived from SMPS measurement* | 309 | 309 | - |

^†^ For details of derivation see Supplementary Information_2.

^††^ *M, dpp & ρ* stand for mass, primary particle size and density.

**Table S4** Additional dose metrics for *in vivo* inhalation studies. ^†^

| Group |  | FeOx-mix  High Dose | Fe_3_O_4_  High Dose | Fe_3_O_4_  Low Dose |
| --- | --- | --- | --- | --- |
| Agglomerate # Dose | Lung (#) | 2.21 X 10^9^ | 2.12 X 10^9^ | 2.12 X 10^8^ |
|  | TB (#/cm^2^) | 1.69 X 10^7^ | 1.61 X 10^7^ | 1.61 X 10^6^ |
| PP # Dose | Lung (#) | 3.33 X 10^12^ | 7.37 X 10^11^ | 7.26 X 10^10^ |
|  | TB (#/cm^2^) | 2.54 X 10^10^ | 5.61 X 10^9^ | 5.53 X 10^8^ |
| Volume Dose | Lung (m^3^) | 2.94 X 10^-12^ | 2.83 X 10^-12^ | 2.78 X 10^-13^ |
|  | TB (m^3^/cm^2^) | 2.24 X 10^-14^ | 2.15 X 10^-14^ | 2.12 X 10^-15^ |
| GSA Dose | Lung (nm^2^) | 1.08 X 10^15^ | 8.73 X 10^14^ | 8.60 X 10^13^ |
|  | TB (nm^2^/cm^2^) | 8.20 X 10^12^ | 6.65 X 10^12^ | 6.55 X 10^11^ |
| ASA Dose | Lung (nm^2^) | 1.91 X 10^14^ | 1.91 X 10^14^ | 1.55 X 10^13^ |
|  | TB (nm^2^/cm^2^) | 1.46 X 10^12^ | 1.46 X 10^12^ | 1.18 X 10^11^ |

^†^ For details of derivation see Supplementary Information_2.

**References**

1. Campbell JA. Effects of Precipitated Silica and of Iron Oxide on the Incidence of Primary Lung Tumours in Mice. British Medical Journal. 1940;2(4156):275.

2. Harding HE, Grout JL, Davies TA. The experimental production of X-ray shadows in the lungs by inhalation of industrial dusts; iron oxide. Br J Ind Med. 1947;4(4):223, 32.

3. Gibb FR, Morrow PE. Alveolar clearance in dogs after inhalation of an iron 59 oxide aerosol. Journal of Applied Physiology. 1962;17(3):429-32.

4. Casarett LJ, Epstein B. Deposition and Fate of Inhaled Iron-59 Oxide in Rats. American Industrial Hygiene Association Journal. 1966;27(6):533-8.

5. Hewitt PJ, editor Deposition and elimination of iron oxide aerosol from the lung of rats: comparison with ICRP predictions for man. . In: Snyder WS, editor, Proceedings of the Third International Congress of the International Radiation Protection Association, Washington, DC, 1974: 1249–54; 1974.

6. Sorokin SP, Brain JD. Pathways of clearance in mouse lungs exposed to iron oxide aerosols. The Anatomical Record. 1975;181(3):581-625.

7. Watson AY, Brain JD. Uptake of iron aerosols by mouse airway epithelium. Lab Invest. 1979;40(4):450-9.

8. Oberdorster G, Green FHY, Freedman AP. Clearance of 59Fe3O4 Particles from the Lungs of Rats During Exposure to Coal Mine Dust and diesel Exhaust. J Aerosol Science 1984;15:235-7.

9. Brain JD, Bloom SB, Valberg PA, Gehr P. Correlation Between the Behavior of Magnetic Iron Oxide Particles in the Lungs of Rabbits and Phagocytosis. Experimental Lung Research. 1984;6(2):115-31.

10. Zhou Y-M, Zhong C-Y, Kennedy IM, Pinkerton KE. Pulmonary responses of acute exposure to ultrafine iron particles in healthy adult rats. Environmental Toxicology. 2003;18(4):227-35.

11. Pettibone JM, Adamcakova-Dodd A, Thorne PS, O'Shaughnessy PT, Weydert JA, Grassian VH. Inflammatory response of mice following inhalation exposure to iron and copper nanoparticles. Nanotoxicology. 2008;2(4):189-204.

12. Martin AR, Thompson RB, Finlay WH. MRI Measurement of Regional Lung Deposition in Mice Exposed Nose-Only to Nebulized Superparamagnetic Iron Oxide Nanoparticles. Journal of Aerosol Medicine and Pulmonary Drug Delivery. 2008;21(4):335-42.

13. Kwon J-T, Hwang S-K, Jin H, Kim D-S, Minai-Tehrani A, Yoon H-J, Choi M, Yoon T-J, Han D-Y, Kang Y-W, Yoon B-I, Lee J-K, Cho M-H. Body Distribution of Inhaled Fluorescent Magnetic Nanoparticles in the Mice. Journal of Occupational Health. 2008;50(1):1-6.

14. Kwon J-T, Kim D-S, Minai-Tehrani A, Hwang S-K, Chang S-H, Lee E-S, Xu C-X, Lim HT, Kim J-E, Yoon B-I, An G-H, Lee K-H, Lee J-K, Cho M-H. Inhaled Fluorescent Magnetic Nanoparticles Induced Extramedullary Hematopoiesis in the Spleen of Mice. Journal of Occupational Health. 2009;51(5):423-31.

15. Pauluhn J. Retrospective analysis of 4-week inhalation studies in rats with focus on fate and pulmonary toxicity of two nanosized aluminum oxyhydroxides (boehmite) and pigment-grade iron oxide (magnetite): The key metric of dose is particle mass and not particle surface area. Toxicology. 2009;259(3):140-8.

16. Pham H, Bonham AC, Pinkerton KE, Chen C-Y. Central neuroplasticity and decreased heart rate variability after particulate matter exposure in mice. Environmental health perspectives. 2009;117(9):1448-53.

17. Zhong C-Y, Zhou Y-M, Smith KR, Kennedy IM, Chen C-Y, Aust AE, Pinkerton KE. Oxidative Injury in The Lungs of Neonatal Rats Following Short-Term Exposure to Ultrafine Iron and Soot Particles. Journal of Toxicology and Environmental Health, Part A. 2010;73(12):837-47.

18. Xie Y, Worth Longest P, Xu YH, Wang JP, Wiedmann TS. <em>In Vitro</em> and <em>In Vivo</em> Lung Deposition of Coated Magnetic Aerosol Particles. Journal of Pharmaceutical Sciences. 2010;99(11):4658-68.

19. Pauluhn J, Wiemann M. Siderite (FeCO 3 ) and magnetite (Fe 3 O 4 ) overload-dependent pulmonary toxicity is determined by the poorly soluble particle not the iron content. Inhalation toxicology. 2011;23:763-83.

20. Pauluhn J. Subchronic inhalation toxicity of iron oxide (magnetite, Fe3O4) in rats: Pulmonary toxicity is determined by the particle kinetics typical of poorly soluble particles2012. 488-504 p.

21. Srinivas A, Rao P, Ganapathy S, Anumolu G, Murthy P, Neelakanta Reddy P. Oxidative stress and inflammatory responses of rat following acute inhalation exposure to iron oxide nanoparticles2012.

22. Sotiriou GA, Diaz E, Long MS, Godleski J, Brain J, Pratsinis SE, Demokritou P. A novel platform for pulmonary and cardiovascular toxicological characterization of inhaled engineered nanomaterials. Nanotoxicology. 2012;6(6):680-90.

23. Sadhukha T, Wiedmann TS, Panyam J. Inhalable magnetic nanoparticles for targeted hyperthermia in lung cancer therapy. Biomaterials. 2013;34(21):5163-71.

24. Teeguarden JG, Mikheev VB, Minard KR, Forsythe WC, Wang W, Sharma G, Karin N, Tilton SC, Waters KM, Asgharian B, Price OR, Pounds JG, Thrall BD. Comparative iron oxide nanoparticle cellular dosimetry and response in mice by the inhalation and liquid cell culture exposure routes. Part Fibre Toxicol. 2014;11:46-.

25. Hofmann T, Ma-Hock L, Strauss V, Treumann S, Rey Moreno M, Neubauer N, Wohlleben W, Gröters S, Wiench K, Veith U, Teubner W, van Ravenzwaay B, Landsiedel R. Comparative short-term inhalation toxicity of five organic diketopyrrolopyrrole pigments and two inorganic iron-oxide-based pigments. Inhal Toxicol. 2016;28(10):463-79.

26. Sutunkova MP, Katsnelson BA, Privalova LI, Gurvich VB, Konysheva LK, Shur VY, Shishkina EV, Minigalieva IA, Solovjeva SN, Grebenkina SV, Zubarev IV. On the contribution of the phagocytosis and the solubilization to the iron oxide nanoparticles retention in and elimination from lungs under long-term inhalation exposure. Toxicology. 2016;363-364:19-28.

27. Hopkins LE, Laing EA, Peake JL, Uyeminami D, Mack SM, Li X, Smiley-Jewell S, Pinkerton KE. Repeated Iron-Soot Exposure and Nose-to-brain Transport of Inhaled Ultrafine Particles. Toxicol Pathol. 2018;46(1):75-84.
